# Underestimation of personal carbon footprint inequality in four diverse countries

**DOI:** 10.1038/s41558-024-02130-y

**Published:** 2024-09-12

**Authors:** Kristian S. Nielsen, Jan M. Bauer, Ramit Debnath, Charles A. Emogor, Sonja M. Geiger, Sakshi Ghai, Wencke Gwozdz, Ulf J. J. Hahnel

**Affiliations:** 1 Department of Management, Society and Communication, Copenhagen Business School, Frederiksberg, Denmark; 2 Cambridge Collective Intelligence and Design Group, University of Cambridge, Cambridge, UK; 3 Climate and Social Intelligence Lab, California Institute of Technology, Pasadena, CA USA; 4 Department of Zoology, University of Cambridge, Cambridge, UK; 5 School of Psychology, Murdoch University, Perth, Australia; 6 Oxford Internet Institute, University of Oxford, Oxford, UK; 7 Department of Consumer Research, Communication and Food Sociology, Justus-Liebig-University Giessen, Giessen, Germany; 8 Faculty of Psychology, University of Basel, Basel, Switzerland

**Keywords:** Climate change, Psychology, Policy, Interdisciplinary studies, Climate-change mitigation

## Abstract

Extensive research highlights global and within-country inequality in personal carbon footprints. However, the extent to which people are aware of these inequalities remains unclear. Here we use an online survey distributed across four diverse countries: Denmark, India, Nigeria and the USA, to show widespread underestimation of carbon footprint inequality, irrespective of participants’ country and income segment. Of the 4,003 participants, within each country, 50% of participants were sampled from the top 10% income group. Our results show links between carbon footprint inequality perceptions and climate policy support, but with significant variations observed across the four countries and with participants’ income segments. Furthermore, there are links to the perceived fairness of actual carbon footprint inequality, highlighting the need to raise awareness about carbon footprint inequality and further unpack its implications for climate justice and policy.

## Abstract (web summary)

Existing studies show carbon footprint inequality between and within countries, but awareness of this inequality is unclear. This study finds widespread underestimation of carbon footprint inequality and its associations with climate policy support and perceived fairness.

## Main

High-income countries are responsible for a disproportionate share of global greenhouse gas (GHG) emissions causing climate change^1–3^. This disparity is mirrored in personal carbon footprints, which are typically substantially higher in wealthier countries^4,5^. For example, when considering GHG embedded in goods, services, and public and private investments, the average personal carbon footprint is 1.6 tonnes of carbon dioxide equivalent (tCO_2_e) in Nigeria compared with 21.1 tCO_2_e in the USA (https://wid.world/data/)^5^. However, such country averages can hide inequalities in personal carbon footprints within countries^6–11^, usually as a function of income and wealth^5,7,12^. Studies show that the footprints of wealthier individuals can be orders of magnitude higher than the country average^5,9,13–15^.

Inequality in personal carbon footprints primarily reflects differences in consumption patterns and associated GHG emissions. For example, on average, wealthier individuals travel more frequently and for longer distances by air^16^, own larger and sometimes multiple homes^7,17^, and have higher GHG emissions from private and work-related vehicle use^7,9,18,19^. Personal carbon footprint accounting traditionally only considers GHG emissions linked to consumption activities^17,20,21^. However, more recent studies have incorporated emissions associated with private investments, such as in stocks, bonds or real estate^5,22^, further allowing assessments of people’s indirect contribution to climate change.

Existing research has documented carbon footprint inequality between and within countries, but the extent to which people are aware of these inequalities remains unclear. Evidence is emerging on people’s perceptions of the carbon footprints of different consumer behaviours, revealing widespread carbon innumeracy^23^ and misperceptions^24–28^. Such discrepancies include overestimating the carbon footprints of lower-impact consumer behaviours (for example, recycling, shutting off the lights and avoiding plastic packaging) and underestimating those of higher-impact behaviours (such as red meat consumption, air travel, and heating and cooling homes)^24,25,29^.

However, there is limited evidence on whether these misperceptions extend to people’s perceptions of the composition and scale of personal carbon footprints and their ability to make inter-individual comparisons^30^. Similar to perceiving economic inequality^31–33^, forming accurate perceptions of carbon footprint inequality requires access to cues of inequality, attending to, comprehending and processing these cues, and the ability to summarize them into a representation of inequality^34^. Forming perceptions of carbon footprint inequality has additional complexity compared with economic inequality by also requiring information about people’s behaviour and the GHG-emissions intensity of this behaviour. Access to and inferences based on this information will depend on numerous factors, including socioeconomic status, social network, media exposure and carbon literacy.

Examining people’s perceptions of carbon footprint inequality is important because inaccurate perceptions may skew perceived responsibilities for mitigating climate change. Moreover, underestimating carbon footprint inequality may undermine support for ambitious climate policies and weaken the perceived importance of inter- and intranational climate justice^35–37^. For example, a recent meta-analysis found public perceptions of distributional fairness to be among the strongest predictors of climate policy support^38^.

In this Article, we investigate perceptions of carbon footprint inequality using a preregistered survey (https://osf.io/8qtfy/) deployed across four heterogeneous countries: Denmark, India, Nigeria and the USA (*n* = 4,003). We selected these countries owing to their differences in carbon footprint inequality, average personal carbon footprints and economic inequality. They also represent considerable geographical, economic, political and cultural diversity, including in their approach to climate policy. As a result, this study increases the representation of diverse countries across the low–middle and high-income segments, thus limiting the well-known WEIRD (white, educated, industrialized, rich and democratic) bias in behavioural science^39,40^. Within each country, we sampled ~1,000 participants equally split across two population segments: people whose personal income belonged to the top 10% income bracket (top 10% sample) and those whose personal income was below the threshold for the top 10% income bracket (general population sample). This sampling strategy allowed us to investigate the perceptions of high-income individuals who, despite their disproportionately large carbon footprints, are systematically underrepresented in survey-based and behavioural science research^7,41^.

Our study has three overarching objectives. First, we examine the accuracy of people’s perceptions of within-country carbon footprint inequality, hypothesizing that people would generally underestimate carbon footprint inequality (H1). Second, we investigate the potential implications of (in)accurate climate footprint inequality perceptions for people’s support for climate change policies, hypothesizing that a stronger underestimation of carbon footprint inequality would be associated with lower climate policy support (H2). Finally, we examine the relationships between perceptions of carbon footprint inequality and the perceived fairness of actual carbon footprint inequality, hypothesizing that a stronger underestimation of carbon footprint inequality would be associated with lower perceived fairness after receiving information on the actual carbon footprint inequality (H3).

## Perceptions of carbon footprint inequality

To measure perceptions of carbon footprint inequality, participants estimated the average personal carbon footprints specific to three income groups (the bottom 50%, the top 10% and the top 1% of income) within their country. In line with the preregistration, we analysed the relative inequality estimations by computing differences in relative estimation errors (estimated versus actual carbon footprint) for the three income groups. For all analyses, we adjusted our significance level to the preregistered, more stringent threshold of *P* = 0.01 owing to multiple hypothesis testing.

**Fig. 1:**
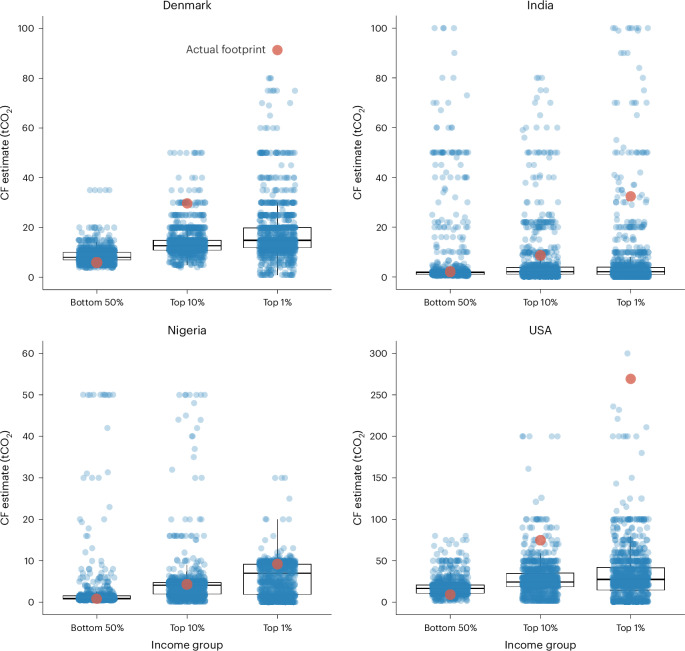
Perceptions of the average personal carbon footprints across income groups. Each dot represents a participant’s estimate of the average carbon footprint (CF) within the respective income group. The red dots show the actual carbon footprint within each income group. The figure only displays carbon footprint estimates within the preregistered 2.5–97.5 percentiles (see Methods). However, additional outliers for India and Nigeria are excluded from the figure to enhance data representation. Similarly, the *y-*axis ranges are adjusted for each country to improve the interpretability of the boxplots (see Supplementary Table 1 for descriptive statistics with the total sample). Sample sizes: Denmark (*N*_bottom 50%_ = 957, *N*_top 10%_ = 952, *N*_top 1%_ = 967), India (*N*_bottom 50%_ = 962, *N*_top 10%_ = 962, *N*_top 1%_ = 976), Nigeria (*N*_bottom 50%_ = 971, *N*_top 10%_ = 962, *N*_top 1%_ = 976) and USA (*N*_bottom 50%_ = 958, *N*_top 10%_ = 967, *N*_top 1%_ = 955). Box, first and third quartiles; central horizontal line, median; upper vertical line end, largest value smaller than 1.5 times the interquartile range; lower vertical line end, smallest value larger than 1.5 times the interquartile range.

Descriptively, most participants across the four countries overestimated the average personal carbon footprint within the bottom 50% of income and underestimated the average footprints within the top 10% and top 1% of income (Fig. 1 and Supplementary Table 1). Accordingly, in the aggregate data, there were significant differences in relative estimation errors between the bottom 50% and both high-income groups for all four countries (dif_B50−T10_: *B* = −2.51, 95% confidence interval (CI) [−2.76, −2.26], *P* < 0.001; dif_B50−T1_: *B* = −2.90, 95% CI [−3.15, −2.65], *P* < 0.001; Supplementary Table 2), thereby supporting H1. This suggests widespread underestimation of the relative carbon footprint differences between income groups. Participants’ own income segment (general population versus top 10% of income within their country) did not explain variance in relative estimation errors (*B* = −0.01, 95% CI [−0.23, 0.21], *P* = 0.922). Complementary models exploring country-level variations showed that the underestimation of carbon footprint inequality differed between the four countries (*F*(6, 7,442) = 66.62, *P* < 0.001). Although evident in all countries, the largest differences in relative estimations were observed in India, where the vast majority of participants overestimated the average carbon footprint within the bottom 50% and underestimated those of the top 10% and top 1% (Supplementary Table 3).

**Extended Data Fig. 1:**
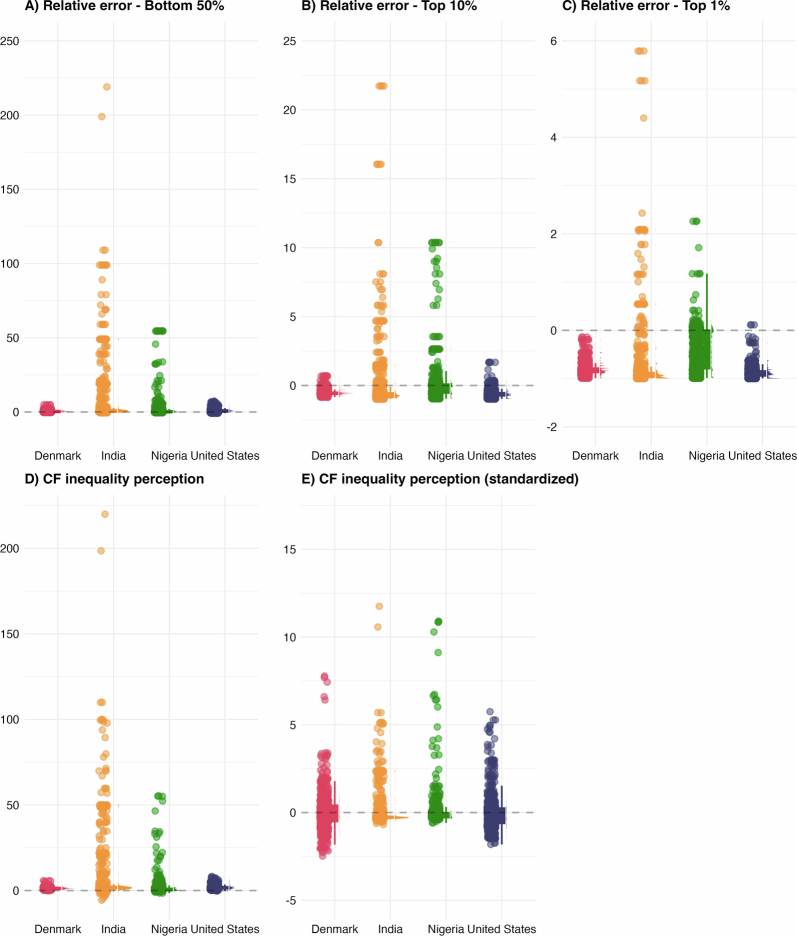
Carbon footprint inequality perception by country. **A**, relative estimation error for the Bottom 50% of income (2.5-97.5 percentiles retained per income group and country as pre-registered; see Methods). **B**, relative estimation error for the Top 10% of income. **C**, relative estimation error for the Top 1% of income. **D**, carbon footprint inequality perception with the pre-registered outlier removal criteria (see Methods). Positive values reflect an underestimation of the average carbon footprint of the Top 1% income group relative to the Bottom 50% income group. In contrast, negative values indicate an overestimation of the average carbon footprint of the Top 1 % income group relative to the Bottom 50% income group. **E**, carbon footprint inequality perception z-standardized at the country level. Box, first and third quartiles; central horizontal line, median; upper vertical line end, largest value smaller than 1.5 times the interquartile range; lower vertical line end, smallest value larger than 1.5 times the interquartile range.

**Extended Data Fig. 2:**
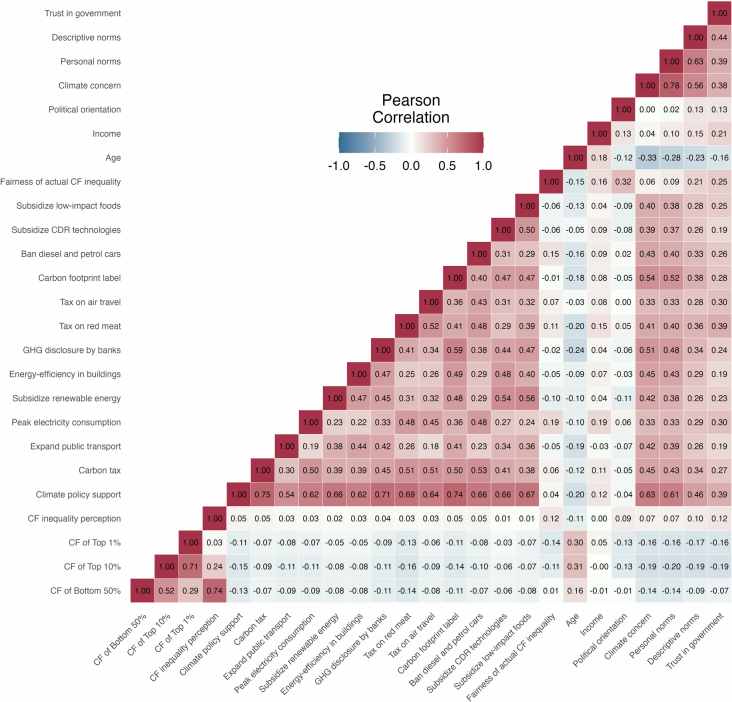
Heat map of bivariate correlations. The heat map includes all relevant outcome variables and socio-demographic and psychological covariates. The estimated personal carbon footprints within the Bottom 50%, Top 10%, and Top 1% are detailed without outliers in accordance with the pre-registration.

To create an overall index of participants’ perception of carbon footprint inequality (hereafter ‘carbon footprint inequality perception’), we computed the preregistered difference between the relative estimation error for the bottom 50% and the top 1% of income (Extended Data Fig. 1). Carbon footprint inequality perception thus reflects the extent to which participants underestimated (or overestimated) the average personal carbon footprints of the top 1% income group relative to the bottom 50% income group, with positive values indicating an underestimation of carbon footprint inequality and negative values an overestimation. Of the participants, 93% had a positive index score, indicating that they underestimated carbon footprint inequality. We provide results from aggregate and country-specific regression models examining which socio-demographic and psychological variables predicted differences in carbon footprint inequality perception in the Supplementary Information (Supplementary Table 3; see Extended Data Fig. 2 for heat map with bivariate correlations).

## Climate policy support

**Fig. 2:**
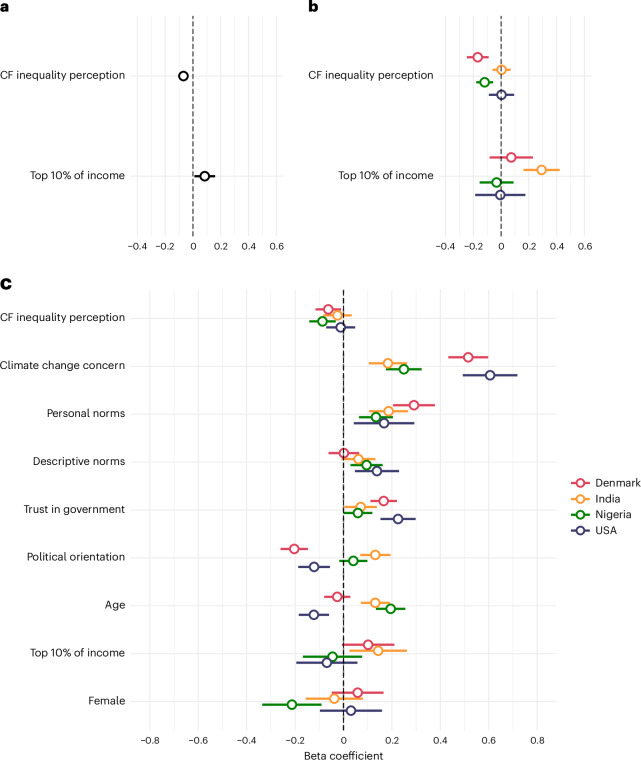
Predictors of composite climate policy support. **a**, Mixed-effects regression model predicting composite climate policy support across Denmark, India, Nigeria and the USA (*N* = 3,756). **b**, Country-specific linear regression models predicting composite climate policy support. Sample sizes: Denmark (*N* = 931), India (*N* = 949), Nigeria (*N* = 956) and the USA (*N* = 920). **c**, Country-specific linear regression models predicting composite climate policy support with socio-demographic and psychological covariates. All predictors were standardized at the country level, except for ‘top 10% of income’ and ‘female’ (see Methods). Top 10% of income shows a coefficient relative to participants belonging to the ‘general population’ in their country, whereas female shows a coefficient relative to identifying as male. Political orientation is coded from left to right, with higher values representing a more right-leaning political orientation. Sample sizes: Denmark (*N* = 923), India (*N* = 949), Nigeria (*N* = 956) and the USA (*N* = 919). The dots in **a**–**c** represent point estimates with 95% confidence intervals.

**Extended Data Fig. 3:**
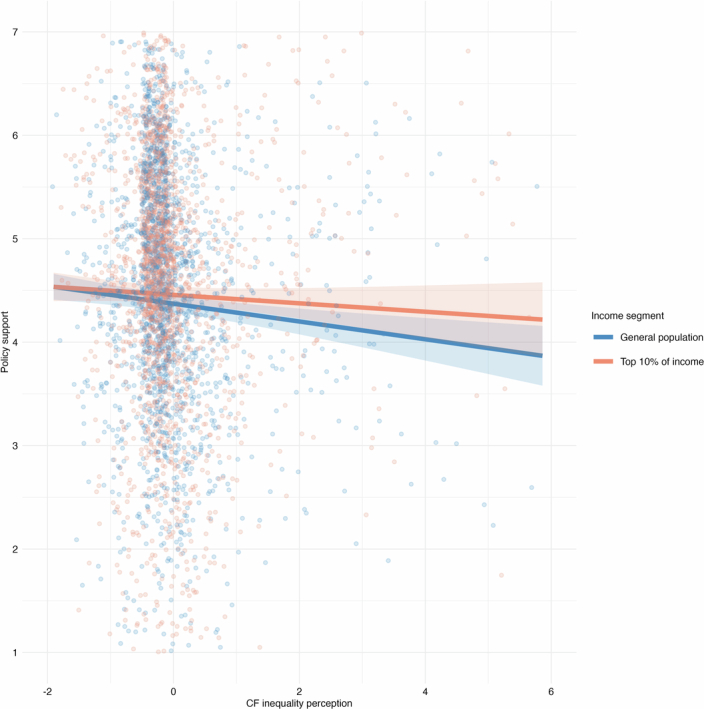
Interaction between carbon footprint inequality perception and participants’ income segment predicting composite climate policy support. Result of linear regression composite climate policy support with an interaction term between carbon footprint inequality perception and participants’ income segment. The regression model included country as a covariate. Carbon footprint inequality perception was standardized at the country level, and the positive values indicate an underestimation of carbon footprint inequality and negative values an overestimation. Lines and shading represent linear regressions and 95% confidence intervals, respectively. The sample size is *N* = 3,756.

We next examined the relationship between carbon footprint inequality perception and climate policy support. Descriptively, we observed a main effect of the country on climate policy support (*F*(3, 3,999) = 138.8, *P* < 0.001), with the lowest support in the USA and the highest support in India. In support of H2, inequality perception was negatively associated with composite climate policy support at the aggregate level (*B* = −0.07; 95% CI [−0.03, −0.11], *P* < 0.001; Fig. 2a and Supplementary Table 4). Thus, the more participants underestimated actual carbon footprint inequality, the less they supported climate policies. Country-specific models only showed statistically significant and negative relationships between carbon footprint inequality perception and climate policy support in Denmark and Nigeria (Fig. 2b and Supplementary Table 5). There was no statistically significant main effect of participants’ own income segment on climate policy support (*B* = 0.08, 95% CI [0.01, 0.16], *P* = 0.025; Fig. 2a and Supplementary Table 4). We also explored an interaction effect between carbon footprint inequality perception and participants’ income segment. However, we found no statistically significant interaction effect at the aggregate level (*B* = 0.05; 95% CI [−0.03, 0.12], *P* = 0.224; Extended Data Fig. 3) or in the country-specific analyses (Supplementary Table 6).

**Extended Data Fig. 4:**
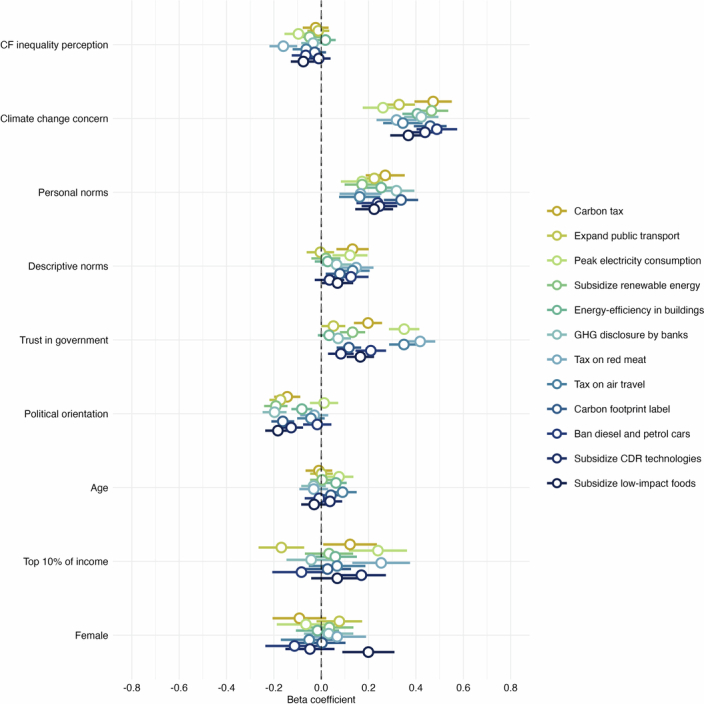
Support for individual climate policies (aggregate). Results of mixed-effects regression models predicting support for individual climate policies. All covariates were standardized at the country level, except ‘Top 10% of income’ and Female (see Methods). Female shows a coefficient relative to identifying as male. The dots represent point estimates with 95% confidence intervals. The sample size is *N* = 3,747 for all policies.

As preregistered, we explored the stability of the relationship between carbon footprint inequality perception and climate policy support when adding socio-demographic and psychological variables to the model. At the aggregate level, carbon footprint inequality perception remained a statistically significant predictor of climate policy support, showing a negative relationship (*B* = −0.05, 95% CI [−0.08, −0.02], *P* < 0.001; Supplementary Table 7). Climate change concern was the strongest predictor of composite climate policy support, with more concerned participants reporting greater support for climate policies (*B* = 0.40, 95% CI [0.36, 0.44], *P* < 0.001). Trust in government, personal norms and descriptive norms also positively predicted composite policy support (Supplementary Table 7). Country-specific regression analyses similarly revealed considerable between-country heterogeneity in the predictiveness of socio-demographic and psychological variables (Fig. 2c and Supplementary Table 8). Policy-specific analyses at the aggregate level further showed that the predictiveness of some variables, such as climate change concern and personal norms, were rather consistent across the climate policies, whereas others, such as trust in government and participants’ income segment, varied substantially across policies (Extended Data Fig. 4; see Supplementary Figs. 1–4 for policy-specific analyses at the country level).

## Perceived fairness of actual carbon footprint inequality

Following the estimation of personal carbon footprints, we informed participants of the actual carbon footprints for the three income groups within their country. They subsequently reported the perceived fairness of the actual differences in carbon footprints between income groups (see Methods). The measurement of perceived fairness of actual carbon footprint thus reflects fairness perceptions after being informed about the actual inequality in one’s own country, distinguishing itself from conventional perceptional measures^42^. On average, participants perceived the actual carbon footprint inequality as slightly unfair (*M*_Total_ = 3.67, s.d. = 1.93; rated from 1–7). However, there were significant mean differences between countries (*F*(3, 3,999) = 71.71, *P* < 0.001), with perceived fairness being lowest in Denmark and the USA.

**Fig. 3:**
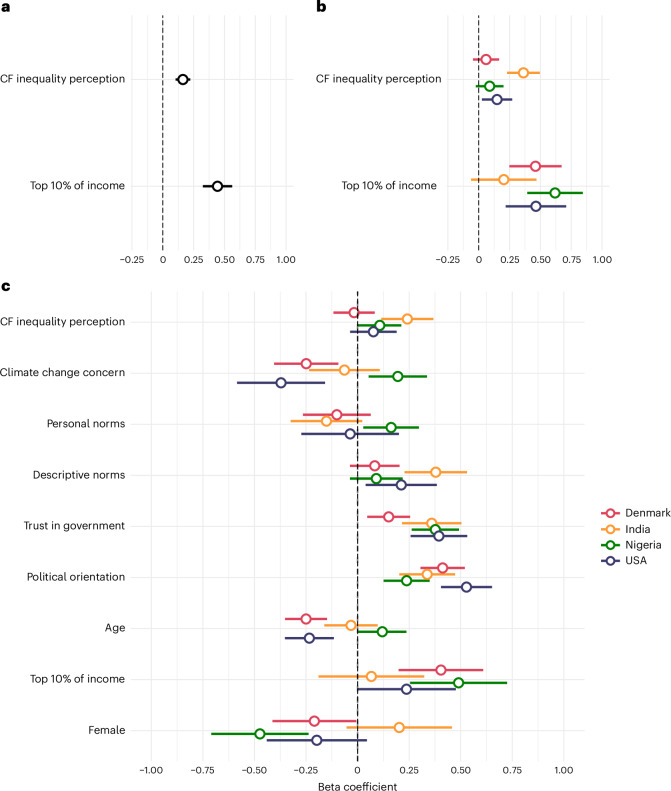
Predictors of the perceived fairness of actual carbon footprint inequality. **a**, Mixed-effects regression model predicting perceived fairness of actual carbon footprint inequality across Denmark, India, Nigeria and the USA (*N* = 3,756). **b**, Country-specific linear regression models predicting perceived fairness of actual carbon footprint inequality. Sample sizes: Denmark (*N* = 931), India (*N* = 949), Nigeria (*N* = 956) and the USA (*N* = 920). **c**, Country-specific linear regression models predicting perceived fairness of actual carbon footprint inequality with socio-demographic and psychological covariates. All predictors were standardized at the country level, except for ‘top 10% of income’ and ‘female’ (see Methods). Top 10% of income shows a coefficient relative to participants belonging to the ‘general population’ in their country, whereas female shows a coefficient relative to identifying as male. Political orientation is coded from left to right, with higher values representing a more right-leaning political orientation. Sample sizes: Denmark (*N* = 931), India (*N* = 949), Nigeria (*N* = 956) and the USA (*N* = 920). The dots in **a**–**c** represent point estimates with 95% confidence intervals.

**Extended Data Fig. 5:**
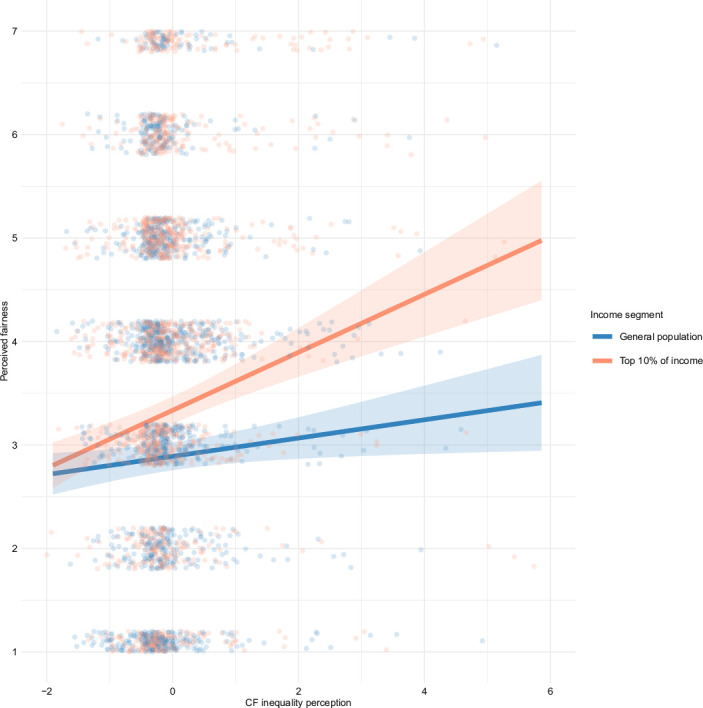
Interaction between carbon footprint inequality perception and participants’ income segment predicting perceived fairness. Result of linear regression predicting the perceived fairness actual carbon footprint inequality with an interaction term between carbon footprint inequality perception and participants’ income segment. The regression model included country as a covariate. Carbon footprint inequality perception was standardized at the country level, and the positive values indicate an underestimation of carbon footprint inequality and negative values an overestimation. Lines and shading represent linear regressions and 95% confidence intervals, respectively. The sample size is *N* = 3,756.

In testing H3, we examined the relationship between carbon footprint inequality perception and perceived fairness of actual carbon footprint inequality. Although we identified a statistically significant relationship, the direction was contrary to our hypothesis (*B* = 0.16, 95% CI [0.10, 0.22], *P* < 0.001; Fig. 3a and Supplementary Table 9): the more participants underestimated carbon footprint inequality, the fairer they perceived the actual inequality. Exploratory analyses showed that this relationship was only evident in India (Fig. 3b and Supplementary Table 10). Across all countries, participants from the top 10% income segment perceived the actual carbon footprint inequality to be significantly fairer than those from the general population (*B* = 0.44, 95% CI [0.32, 0.56], *P* < 0.001; Fig. 3a). To unpack this further, we explored an interaction effect between participants’ carbon footprint inequality perception and their income segment. At the aggregate level, we observed a statistically significant interaction effect (*B* = 0.19, 95% CI [0.07, 0.31], *P* = 0.002; Supplementary Table 11 and Extended Data Fig. 5). This suggests that participants from the top 10% of income primarily drive the positive relationship between perceived fairness of actual carbon footprint inequality and inequality perception. However, this interaction was not statistically significant in the country-specific analyses, which may reflect the lower statistical power to detect such an effect due to the smaller sample sizes (Supplementary Table 11).

We next tested relationships between socio-demographic and psychological factors and the perceived fairness of carbon footprint inequality. At the aggregate level, the relationship between carbon footprint inequality perception and perceived fairness of footprint inequality remained stable, even with the introduction of socio-demographic and psychological factors (*B* = 0.10, 95% CI [0.05, 0.16], *P* < 0.001; Supplementary Table 12). Participants with a more right-leaning political orientation, higher trust in the government, higher perceived descriptive norms and lower climate change concern reported higher perceived fairness of actual carbon footprint inequality. Younger and male participants also tended to perceive actual carbon footprint inequality as fairer (Supplementary Table 12). However, exploratory country-specific analyses revealed noteworthy heterogeneity in some of these relationships (Fig. 3c and Supplementary Table 13).

## Discussion

Extensive research has documented profound inequality in personal carbon footprints between and within countries. In this study, we investigated perceptions of within-country carbon footprint inequality across four socioeconomically heterogeneous countries. Our results reveal a widespread underestimation of personal carbon footprint inequality, including among the wealthiest population segments, and that such underestimation may translate into lower climate policy support and higher perceived fairness of carbon footprint inequality, even after revealing actual inequality in personal carbon footprints.

We observed the largest absolute underestimations of personal carbon footprints for the top 10% and top 1% income groups in Denmark and the USA, probably reflecting these income groups’ substantially higher absolute footprints compared with the country average. Owing to the complexity of estimating personal carbon footprints and the variation across footprinting methodologies, we focused our analyses on relative estimations of carbon footprint inequality. On the basis of differences in carbon footprint estimations between the bottom 50% and the top 1% income groups, this analysis revealed the largest differences in relative estimations in India. Our results strongly support H1 and extend existing studies using objective indicators of carbon footprint inequality to psychologically relevant subjective representations of these inequalities.

Notably, there was no difference in carbon footprint inequality perceptions between participants from the top 10% income segment and those with an income below this threshold (labelled the ‘general population’). This finding suggests that underestimations of carbon footprint inequality are apparent across socioeconomic groups and thus unlikely to reflect motivated perceptions. A motivated-reasoning account would predict larger underestimations in segments with larger personal carbon footprints, such as this study’s top 10% income segment.

Does people’s perception of carbon footprint inequality matter for climate change mitigation objectives? We addressed this question by examining the relationship between carbon footprint inequality perception and climate policy support. At the aggregate level, we observed a negative relationship between carbon footprint inequality perception and support for a composite measure of 12 climate policies, suggesting that participants who underestimated carbon footprint inequality more were less supportive of climate policies. Country-specific analyses showed that our samples from Denmark and Nigeria primarily drove this effect. Hence, we observe mixed support for H2, partially contrasting previous work^43^.

This study’s unique representation of high-income individuals is especially interesting when examining support for climate policies. Unexpectedly, at the aggregate level, participants from the top 10% of income reported stronger support for climate policies. However, this effect was only significant in India, potentially reflecting greater access to information and interest in policy among individuals from the top 10% of income. Studies have also shown that the level of education in India is positively related to different pro-environmental behaviours^44^. Regarding support for specific policies, participants from the top 10% of income reported stronger support for increasing the price of electricity during peak times, implementing a tax on red meat and subsidizing carbon dioxide removal technologies. Their stronger support for exactly these policies might reflect their greater capacity to absorb price increases and a stronger endorsement of technological solutionism. Conversely, participants from the general population reported stronger support for expanding public transport. Importantly, these results reflect policy-specific analyses at the aggregate level and therefore shield country differences as depicted in Supplementary Figs. 1–4.

Accurately perceiving personal carbon footprint inequality is challenging. But how fair is carbon footprint inequality perceived once learning about the actual inequality? On average, participants perceived the actual inequality as slightly unfair but with considerable variation within and between countries. The perceived fairness of actual carbon footprint inequality was lowest in Denmark and the USA, probably reflecting the larger absolute carbon footprints of the high-income groups there. The more participants underestimated carbon footprint inequality, the fairer they perceived the actual inequality, even after receiving information on actual carbon footprint inequality. This finding suggests that participants’ fairness perceptions primarily reflected their initial perceptions of carbon footprint inequality before receiving information about the actual inequality rather than the difference between their initial perceptions and the actual footprints. However, this aggregate relationship between carbon footprint inequality perception and perceived fairness was especially driven by the Indian sample. We thus find limited support for H3.

Interestingly, participants from the top 10% income segment perceived the actual carbon footprint inequality as significantly fairer than those from the general population, except in India. This perceptual difference might reflect a self-serving attitude, whereby participants presumed to have larger personal carbon footprints justify the fairness of within-country carbon footprint inequality.

Our study has several limitations. First, owing to data unavailability, participants were not provided specific income cut-offs when estimating the carbon footprints for the different groups. This may have introduced estimation noise, as participants’ perceptions of the income thresholds could have varied. Second, our deliberate choice to focus on the general population and the top 10% income groups has implications for the representativeness of our sample. We deliberately traded off generalizability for a higher representation of high-income individuals who generally have higher personal carbon footprints and stronger political, organizational and social influence^45,46^. Such a trade-off allowed us to contribute the perspectives of the wealthiest individuals, a segment that remains systematically underrepresented in scientific research^7,41^. Third, there were pronounced outliers in carbon footprint inequality perception in India and Nigeria (Extended Data Fig. 1), yet we decided to maintain our preregistered outlier cut-off. We conducted supplementary analyses with additional outlier removals (±3 s.d.), detailed in Supplementary Tables 14 and 15 and Figs. 5–7, that show the stability of our main results. Fourth, despite following conventional measurement practices^47^, the measure of climate policy support was imperfect, given the complexity of assessing support across highly diverse political and policy landscapes. Relatedly, we cannot account for differences in participants’ knowledge of climate policy (for example, as a function of cultural background or interest in climate change), which may moderate the relationship between perceived carbon footprint inequality and climate policy support. Fifth, we assessed carbon footprint inequality perceptions using a single overall index and perceived fairness of actual carbon footprint inequality with one item only, which may have affected the reliability of the measurements^48^. Finally, although we performed analyses to examine the stability of our main results by introducing relevant socio-demographic and psychological factors to the models, we cannot rule out the possibility that the results suffer from additional confounding.

The carbon footprint estimates used in this study differ from traditional territorial or consumption-based methods by accounting for GHG emissions embedded in public and private investments. Accounting for private investments widens the disparity in personal carbon footprints between income groups, as wealthier individuals often possess larger investment portfolios and consequently generate more GHG emissions. While we consider this a methodological strength, it may have heightened the potential for inaccuracies in participants’ estimates for the high-income groups. Nonetheless, we carefully introduced participants to the accounting method and thoroughly checked their comprehension (see Methods). Furthermore, our analyses focused on relative rather than absolute inequality perceptions, which should have reduced the impact of potentially skewed inaccuracies due to the footprinting methodology.

Despite its limitations, this study has important implications for climate change mitigation research and practice. Our findings underscore the urgent need to raise awareness about carbon footprint inequality to initiate public debates about its fairness and increase attention to social justice concerns in climate policy. While our study cannot identify the most promising strategies for realizing such objectives, future research may uncover which strategies best promote climate justice in practice and policy in different cultures and for different target audiences. Our study does show that correcting carbon footprint inequality perceptions probably did not reduce the perceived fairness of inequality, as underestimating carbon footprint inequality remained positively related to perceived fairness, even after providing information on actual carbon footprint inequality. This finding may dampen expectations that correcting perceptions of within-country carbon footprint inequality will substantially increase climate policy support^49^. However, future studies that experimentally correct carbon footprint inequality perceptions are needed to more comprehensively evaluate their impacts on support for climate policies and other important mitigation objectives.

## Methods

An online survey was conducted in four countries: Denmark, India, Nigeria and the USA in May and June 2023. The survey was developed and administered in English, except for Denmark, where the survey was administered in Danish. In India, to ensure sufficient English comprehension, as Hindi is also widely spoken in the country, only participants who felt comfortable answering in English completed the survey. The survey design, materials, data exclusion criteria and analytical strategy were preregistered via the Open Science Framework (OSF; https://osf.io/8qtfy/).

### Participants

Participants were recruited via the market research companies Nielsen (Denmark and Nigeria) and Qualtrics (India and the USA) and received financial compensation. A total of 4,003 participants completed the survey with the following country breakdown: Denmark (*n* = 1,001), India (*n* = 1,001), Nigeria (*n* = 1,001) and the USA (*n* = 1,000). In each country, the sampled participants were equally split across two income groups: participants whose personal income fell into the top 10% income bracket (top 10% segment) and participants whose personal income was below the threshold for the top 10% income bracket (general population segment). We applied the following income thresholds for the top 10%^50–52^: Denmark (DKK650,000), India (I₹300,000), Nigeria (N₦35,000,000) and the USA (US$130,000) to account for the within-country differences in income level.

For ethical reasons and to ensure high data quality, participants were automatically screened out if one or more of the following preregistered criteria were met: (1) reported being under 18 years old; (2) felt uncomfortable answering the survey in English (only in India); (3) reported ‘prefer not to answer’ on the income question; (4) failed the attention check; (5) answered the comprehension check for the concept of personal carbon footprint wrongly twice. In addition, participants who were probably bots based on Recaptcha score (*n* = 10) or completed the survey unreasonably quickly (*n* = 180) or more than once (*n* = 1) were excluded and replaced with other responses by Nielsen and Qualtrics (not preregistered). The mean age in the full sample was 42.35 (s.d. = 16.59), and 56.8% identified as male, 42.8% as female, 0.3% as non-binary and 0.01% preferred not to say. Supplementary Table 1 presents detailed socio-demographic information for each sample.

### Measures

In line with our research objectives, we collected data on three outcome variables and several psychological and socio-demographic covariates, which are detailed below. Supplementary Table 1 details the descriptive statistics and Extended Data Fig. 2 displays a heat map of bivariate correlations. A full survey overview is available via the preregistration on OSF.

#### Carbon footprint inequality perception

We used carbon footprint data from the World Inequality Database (https://wid.world/data/) to assess perceptions of carbon footprint inequality. These resources offer disaggregated personal carbon footprint data across income groups while accounting for GHG emissions associated with public services and private investments. The country-specific carbon footprint estimates used were the following:

- **Denmark**: bottom 50% (6.0 tCO_2_-eq.), top 10% (29.7 tCO_2_-eq.), top 1% (93.1 tCO_2_-eq.) and country average (10.9 tCO_2_-eq.)
- **India**: bottom 50% (1.0 tCO_2_-eq.), top 10% (8.8 tCO_2_-eq.), top 1% (32.4 tCO_2_-eq.) and country average (2.2 tCO_2_-eq.)
- **Nigeria**: bottom 50% (0.9 tCO_2_-eq.), top 10% (4.4 tCO_2_-eq.), top 1% (9.2 tCO_2_-eq.) and country average (1.6 tCO_2_-eq.)
- **USA**: bottom 50% (9.7 tCO_2_-eq.), top 10% (74.7 tCO_2_-eq.), top 1% (269.3 tCO_2_-eq.) and country average (21.1 tCO_2_-eq.)

In the survey, participants were first introduced to the concept of a personal carbon footprint and the details of the present measurement approach^5^ (see preregistration for specific wording). They then answered a comprehension check to ensure a sufficient comprehension of the concept and the present measurement approach: those who answered wrongly on the first attempt were again presented with the carbon footprint description and allowed to re-answer the comprehension question. They were automatically screened out of the survey if they answered wrongly again.

Next, participants estimated the average personal carbon footprints specific to three income groups within their own country (reflecting the structure of ref. ^5^): the bottom 50% of income, the top 10% of income and the top 1% of income. To assist participants’ estimations and to avoid extreme values^53^, we informed participants of the average personal carbon footprint within their country; for example: “The average Indian has a personal carbon footprint of 2.2 tonnes CO_2_-eq./per year. Remember this means that some Indians will have a lower personal carbon footprint than this number, whereas other Indians will have a higher footprint.”

Participants then estimated the average personal carbon footprint specific to the three income groups, starting with the bottom 50% of income using the following instruction: “We now want you to imagine only the 50% of [country inhabitants] with the lowest income. This means the [country inhabitants] whose income falls within the bottom half of the income distribution. What is the average carbon footprint of a person belonging to this income group?” Similar tailored instructions were presented for the top 10% and top 1% income groups alongside reminders of the average personal carbon footprint in the country. For each estimation, shown on separate pages, participants indicated the perceived personal carbon footprint in CO_2_-equivalents per year. The range of possible values was bounded to prevent extreme values (min = 0.1 tCO_2_e; max = 2,000 tCO_2_e). They also indicated their confidence in each estimate: “How certain are you that your answer is correct?” (1 = not at all certain, to 7 = absolutely certain).

#### Climate policy support

Participants indicated their support for 12 prospective climate policies inspired by recent research^47^. Given their heterogeneous political systems and policy landscapes, we carefully phrased the policies to ensure applicability across the four countries. The instructions read: “Many countries have introduced new policies to reduce the risk of climate change. This includes policies that require or create incentives for reductions in greenhouse gas emissions across domains and actors. How much do you support or oppose adopting the following policies in [country]?” Responses were assessed on a 7-point Likert scale (1 = strongly oppose, 7 = strongly support). The policies were: (1) increase or introduce taxes on products and services that are made from or use fossil fuels (for example, coal, oil, gas); (2) expand public transport (buses, trams, trains); (3) increase the price of electricity consumption during peak times; (4) increase subsidies for renewable energy projects (for example, wind and solar energy); (5) strengthen requirements for energy efficiency in buildings; (6) mandate banks and investment companies to reveal their greenhouse gas emissions to consumers; (7) increase or introduce taxes on red meat (for example, beef, lamb, veal); (8) increase or introduce taxes on air travel; (9) introduce a mandatory carbon footprint label on consumer products; (10) ban the sale of diesel and petrol-engine cars; (11) increase subsidies for technologies that remove greenhouse gases from the atmosphere; and (12) increase subsidies for food products with low greenhouse gas emissions (for example, fruit, vegetables, legumes, cereals). The composite scale showed high internal consistency (Cronbach’s *α* = 0.89).

#### Perceived fairness of actual carbon footprint inequality

After the carbon footprint task, participants were informed about the actual personal carbon footprints within the three income groups (participants could not change their previous responses). They then answered: “How fair or unfair are the differences in personal carbon footprints between income groups?” (1 = not at all fair, 7 = extremely fair).

#### Covariates

Climate change concern was assessed via two items from ref. ^54^: “How worried are you about climate change?” (1 = not at all worried, 7 = extremely worried) and “How important is the issue of climate change to you personally?” (1 = not at all important, 7 = extremely important). Internal consistency was high (Cronbach’s *α* = 0.92). Personal norms were measured using one item: “I feel a personal responsibility to take action to tackle climate change” (1 = strongly disagree, 7 = strongly agree). Descriptive norms were similarly measured using one item: “People in my social group are taking action to tackle climate change” (1 = strongly disagree, 7 = strongly agree). Trust in government was assessed with one item asking: “How trustworthy do you find your national government?” (1 = not at all trustworthy, 7 = extremely trustworthy).

Socio-demographic factors measured were age (in years), gender (male, female, non-binary and ‘prefer not to say’), highest level of education (1 = no schooling completed, 7 = masters degree or above), political orientation^55^ (1 = left, 7 = right) and political party affiliation (we provided all electable parties in each of the countries). To gather data on income, we asked “What was your total annual personal income before taxes in 2022?” on a 10-point scale (plus a ‘prefer not to answer’ option) adapted to the country. Here, response option 10 was the income threshold for being in the top 10% of income in the country (for example, “₹300,000 or more”). Participants who reported an income belonging to the top 10% were subsequently asked another 6-point income question to assess their personal income more accurately: “You indicated that your total annual personal income before taxes in 2022 was more than [top 10% income threshold]. Please select the band that most precisely describes your income.”

### Analytical strategy

In line with the preregistration, we assessed relative differences between estimated and actual personal carbon footprints within three income groups (bottom 50%, top 10% and top 1%) to examine perceptions of carbon footprint inequality.

First, as preregistered, we calculated the relative estimation error per income group by subtracting the true value from each participant’s estimated value and then dividing the derived number by the true value. The relative estimation error was expected to be negative for the top 1% and top 10% income groups, indicating an underestimation of their actual carbon footprints, but positive for the bottom 50% income group, indicating an overestimation of actual emissions in this population group. As preregistered, we removed outliers from relative estimation error calculations (2.5–97.5 percentiles retained per income group and country). Accordingly, we report the main results without outliers but provide descriptive statistics and sensitivity analyses using the full sample and more restrictive outlier removal in the Supplementary Information (Supplementary Tables 2, 4, 9, 14 and 15, and Figs. 5–7).

Second, we calculated the differences in relative estimation error for the bottom 50% income group and the top 1% income group to obtain an overall measure of carbon footprint inequality perception, reflecting participants’ perceptions of the differences in carbon footprints between income groups. Positive values reflect an underestimation of the average carbon footprint of the top 1% income group relative to the bottom 50% income group. By contrast, negative values indicate an overestimation of the average carbon footprint of the top 1 % income group relative to the bottom 50% income group. We then used this index as a predictor of the effects of carbon footprint inequality perception on climate policy support and perceived fairness of actual carbon footprint inequality. A separate analysis using the top 10% income group as a reference instead of the top 1% income group is presented in Supplementary Tables 4 and 9.

To test H1 (that people would generally underestimate carbon footprint inequality), we fitted the preregistered mixed-effects model with relative estimation error as the dependent variable and estimated income group (bottom 50%, top 10% and top 1%) as well as participants’ income segment (top 10% of income versus general population) as fixed-effect predictors. Following our preregistration, we specified participant ID and country as crossed random effects (note that our specification is synonymous with a nested random effect model, which is more appropriate given our data structure, because each participant had a unique ID). To explore the effect of country on carbon footprint inequality perception, we fitted a complementary model with participants’ income segment, estimated income group and country as fixed effects (with an interaction between country and estimated income group) and participant ID as a random effect. We also fitted exploratory multiple linear regression models with covariates at the aggregate and country-specific levels.

To test H2 (that a stronger underestimation of carbon footprint inequality would be associated with lower climate policy support), we fitted the preregistered mixed-effects model with climate policy support as the dependent variable and carbon footprint inequality perception index and participants’ own income segment as fixed-effect predictors, with country used as a random effect. As preregistered, to explore how climate policy support differed across countries, we fitted a complementary model that had country, carbon footprint inequality perception and participants’ income segment as fixed effects, with an interaction between country and carbon footprint inequality perception. We also fitted exploratory multiple linear regression models with covariates at the aggregate and country-specific levels and with an interaction term between carbon footprint inequality perception and participants’ income segment.

To test H3 (that a stronger underestimation of carbon footprint inequality would be associated with lower perceived fairness of actual carbon footprint inequality), we fitted the preregistered mixed-effects model with the perceived fairness of actual carbon footprint inequality as the dependent variable and the carbon footprint inequality perception index and participants’ own income segment as fixed-effect predictors, with country specified as a random effect. Again, we fitted a complementary model that had country, carbon footprint inequality perception and participants’ income segment as fixed effects, with an interaction between country and carbon footprint inequality perception. We also fitted exploratory multiple linear regression models with covariates at the aggregate and country-specific levels and with an interaction term between carbon footprint inequality perception and participants’ income segment.

The following variables were *z*-standardized at the country level (not preregistered) when used as predictors in the mixed-effects and multiple regression analyses: carbon footprint inequality perception, perceived fairness of actual carbon footprint inequality, age, climate change concern, personal norms, descriptive norms and trust in government.

Finally, to account for the increased likelihood of type I errors due to multiple hypothesis testing, we adjusted our significance level to the preregistered, more stringent threshold of *P* = 0.01 for all analyses.

### Deviations from preregistration

To reduce the manuscript’s complexity, we removed a preregistered hypothesis and analyses of perceived behavioural plasticity. This means that H4 in the preregistration became H3 in the manuscript. All results relating to perceived behavioural plasticity are available upon request. We slightly rephrased the hypotheses without changing their meaning. For H2 and H3, we removed (1|ResponseID) from the mixed-effects models because this specification was included by mistake. The standardization of the continuous variables at the country level was not preregistered.

### Reporting summary

Further information on research design is available in the Nature Portfolio Reporting Summary linked to this article.

## Online content

Any methods, additional references, Nature Portfolio reporting summaries, source data, extended data, supplementary information, acknowledgements, peer review information; details of author contributions and competing interests; and statements of data and code availability are available at 10.1038/s41558-024-02130-y.

## Supplementary information

Supplementary Information Supplementary Tables 1–15 and Figs. 1–7.

Reporting Summary

## Data Availability

The data and study materials are available via the Open Science Framework^56^. The personal carbon footprint data were extracted from the World Inequality Database in December 2023 (https://wid.world/data/).

## Code availability

The code is available via the Open Science Framework^56^.
