# Supplementary material for: Underestimation of personal carbon footprint inequality in four diverse countries
Source: Nat Clim Chang. 2024 Sep 12;14(11):1136–43. doi: 10.1038/s41558-024-02130-y (PMC11537954; doi:10.1038/s41558-024-02130-y)

nature climate change

Supplementary information https://doi.org/10.1038/s41558-024-02130-y

Underestimation of personal carbon footprint inequality in four diverse countries

In the format provided by the authors and unedited

Table of contents

Supplementary Table 1 2
Supplementary Table 2 5
Supplementary Table 3 6
Supplementary Table 4 7
Supplementary Table 5 8
Supplementary Table 6 9
Supplementary Table 7 10
Supplementary Table 8 11
Supplementary Table 9 12
Supplementary Table 10 13
Supplementary Table 11 14
Supplementary Table 12 15
Supplementary Table 13 16
Supplementary Table 14 17
Supplementary Table 15 18
Supplementary Fig. 1 19
Supplementary Fig. 2 20
Supplementary Fig. 3 21
Supplementary Fig. 4 22
Supplementary Fig. 5 23
Supplementary Fig. 6 24
Supplementary Fig. 7 25

Supplementary Table 1. Descriptive statistics. Statistics for continuous variables are reported with mean, standard deviation, and median, whereas categorical variables are reported as percentages.

	Denmark				India				Nigeria				United States			
	N	Mean	SD	Median	N	Mean	SD	Median	N	Mean	SD	Median	N	Mean	SD	Median
**Socio-demographics**																
Age	1,001	52.80	15.42	53	1,001	31.11	9.62	29	1,001	35.43	10.51	34	1,000	50.07	17.85	48
Income group	1,001				1,001				1,001				1,000			
… General population	501	50%			501	50%			500	50%			500	50%		
… Top 10% of income	500	50%			500	50%			501	50%			500	50%		
Income (15 categories)	1,001	8.27	3.33	9.00	1,001	9.01	4.34	9.00	1,001	8.00	4.66	10.00	1,000	8.32	3.66	9.50
Gender	1,001				1,001				1,001				1,000			
... Male	540	54%			587	59%			738	74%			408	41%		
... Female	460	46%			411	41%			261	26%			583	58%		
... Non-binary / third gender	1	0.1%			2	0.2%			1	0.1%			9	0.9%		
... Prefer not to say	0	0%			1	0.1%			1	0.1%			0	0%		
Education	1,001				1,001				1,001				1,000			
... No schooling completed	1	0.1%			1	0.1%			1	0.1%			3	0.3%		
... Primary school	3	0.3%			3	0.3%			1	0.1%			6	0.6%		
... Lower secondary school	51	5%			7	0.7%			9	0.9%			9	0.9%		
... Vocational degree	241	24%			20	2%			32	3%			59	6%		
... High school	89	9%			59	6%			149	15%			221	22%		
... College degree	303	30%			487	49%			599	60%			428	43%		
... Master's degree or above	313	31%			424	42%			210	21%			274	27%		
Political orientation (left-right)	993	4.17	1.51	4	1,001	4.91	1.55	5	1,001	4.95	1.60	5	998	4.25	1.75	4.00

**Carbon footprint perceptions**																
CF estimate – Bottom 50%	1,001	13.21	61.43	8.00	1,001	38.71	215.28	2.00	1,001	12.46	111.63	1.00	1,000	32.64	137.25	17.00
CF estimate – Top 10%	1,001	21.50	97.42	12.90	1,001	35.45	203.19	2.20	1,001	17.07	133.07	4.20	1,000	44.37	146.20	25.00
CF estimate – Top 1%	1,001	26.60	102.33	15.00	1,001	34.30	197.43	2.20	1,001	15.58	104.43	7.50	1,000	56.86	178.24	28.00
CF estimate – Bottom 50% (without preregistered outliers)	957	8.92	3.29	8.00	962	6.97	18.20	2.00	971	2.21	5.99	1.00	958	18.53	10.71	17.00
CF estimate – Top 10% (without preregistered outliers)	952	14.37	6.35	12.90	962	7.69	19.85	2.20	962	4.56	6.17	4.20	967	28.71	22.30	25.00
CF estimate – Top 1% (without preregistered outliers)	967	18.13	11.97	15.00	976	8.16	23.05	2.20	976	5.98	4.05	7.00	955	34.22	34.59	28.00
Relative error – Bottom 50%	1,001	1.20	10.24	0.33	1,001	37.71	215.28	1.00	1,001	12.84	124.03	0.11	1,000	2.37	14.15	0.75
Relative error – Top 10%	1,001	-0.28	3.28	-0.57	1,001	3.03	23.09	-0.75	1,001	2.88	30.24	-0.045	1,000	-0.41	1.96	-0.67
Relative error – Top 1%	1,001	-0.71	1.10	-0.84	1,001	0.059	6.09	-0.93	1,001	0.69	11.35	-0.18	1,000	-0.79	0.66	-0.90
Relative error – Bottom 50% (without preregistered outliers)	957	0.49	0.55	0.33	962	5.97	18.20	1.00	971	1.46	6.66	0.11	958	0.91	1.10	0.75
Relative error – Top 10% (without preregistered outliers)	952	-0.52	0.21	-0.57	962	-0.13	2.26	-0.75	962	0.037	1.40	-0.045	967	-0.62	0.30	-0.67
Relative error – Top 1% (without preregistered outliers)	967	-0.81	0.13	-0.84	976	-0.75	0.71	-0.93	976	-0.35	0.44	-0.24	955	-0.87	0.13	-0.90
Carbon footprint inequality perception	1,001	1.92	9.70	1.20	1,001	37.65	212.27	1.89	1,001	12.15	119.04	0.48	1,000	3.15	14.03	1.60
Carbon footprint inequality perception (without preregistered outliers)	931	1.30	0.58	1.20	949	6.75	18.13	1.89	956	1.42	4.94	0.48	920	1.81	1.12	1.65
**Climate policy support**																
Climate policy support (composite)	1,001	4.40	1.23	4.42	1,001	5.06	1.04	5.08	1,001	4.73	0.97	4.75	1,000	4.04	1.41	4.17
Carbon tax	1,001	4.23	1.78	4	1,001	4.60	1.92	5	1,001	4.48	1.84	5	1,000	3.60	2.07	4.00

Expand public transport	1,001	4.98	1.72	5	1,001	6.05	1.38	7	1,001	5.66	1.49	6	1,000	5.09	1.64	5.00
Increase price of peak electricity consumption	1,001	3.12	1.79	3	1,001	3.76	2.05	4	1,001	4.08	2.09	4	1,000	3.05	1.94	3.00
Subsize renewable energy	1,001	5.50	1.55	6	1,001	5.89	1.53	7	1,001	5.18	1.71	5	1,000	4.85	1.92	5.00
Strengthen energy efficiency requirements in buildings	1,001	5.20	1.45	5	1,001	5.74	1.40	6	1,001	5.50	1.48	6	1,000	5.04	1.70	5.00
Mandate GHG disclosure by banks and investment companies	1,001	4.21	1.80	4	1,001	5.46	1.56	6	1,001	5.14	1.65	5	1,000	4.31	1.96	4.00
Tax on red meat	1,001	3.25	2.00	3	1,001	4.64	2.10	5	1,001	3.82	1.92	4	1,000	2.88	1.96	2.00
Tax on air travel	1,001	4.30	1.95	4	1,001	4.19	1.97	4	1,001	4.13	1.87	4	1,000	3.26	1.98	3.00
Introduce mandatory carbon footprint label	1,001	4.46	1.71	4	1,001	5.48	1.55	6	1,001	5.04	1.62	5	1,000	4.19	1.94	4.00
Ban diesel and petrol cars	1,001	3.27	1.95	3	1,001	3.97	2.03	4	1,001	3.82	2.16	4	1,000	2.94	1.96	3.00
Subsidize CDR technologies	1,001	5.39	1.49	6	1,001	5.44	1.79	6	1,001	5.15	1.66	5	1,000	4.80	1.86	5.00
Subsidize low-impact foods	1,001	4.85	1.71	5	1,001	5.52	1.66	6	1,001	4.80	1.84	5	1,000	4.51	1.93	5.00
**Perceived fairness of actual carbon footprint inequality**	1,001	3.12	1.65	3	1,001	4.01	2.11	4	1,001	4.17	1.80	4	1,000	3.37	1.91	3.00
**Psychological**																
Climate change concern	1,001	4.43	1.48	4.50	1,001	6.15	1.14	6.50	1,001	5.53	1.34	5.50	1,000	4.55	1.92	5.00
Personal norms	1,001	4.53	1.69	5	1,001	6.02	1.26	7	1,001	5.56	1.34	6	1,000	4.37	1.94	5.00
Descriptive norms	1,001	4.01	1.46	4	1,001	5.28	1.58	6	1,001	4.86	1.59	5	1,000	3.81	1.84	4.00
Trust in government	1,001	3.57	1.58	4	1,001	5.21	1.64	5	1,001	3.96	2.06	4	1,000	3.29	1.88	3.00

Supplementary Table 2. Relative estimation error associated with estimated personal carbon footprints (test of hypothesis 1).

	**Without outliers (pre-registered)**		**Full sample**	
	Estimates	*95% CI*	*Estimates*	*95% CI*
(Intercept)	2.21 ***	1.42, 3.01	14.65 ***	8.83, 20.47
Estimation error (Top 1%)	-2.90 ***	-3.15, -2.65	-13.72 ***	-16.77, -10.67
Estimation error (Top 10%)	-2.51 ***	-2.76, -2.26	-12.23 ***	-15.27, -9.18
Income segment (Top 10%)	-0.01	-0.23, 0.21	-2.25	-5.10, 0.60
**Random Effects**				
σ^2	31.37		4,841.87	
τ_{00}	1.71 ResponseId		503.16 ResponseId	
	0.61 country		27.80 country	
ICC	0.07		0.10	
N	4 country		4 country	
	3,970 ResponseId		4,003 ResponseId	
Observations	11,565		12,009	
Marginal R^2 / Conditional R^2	0.047 / 0.112		0.007 / 0.105	

Income segment (Top 10%) refers to participants belonging to the Top 10% of income within their country and shows coefficients relative to belonging to the general population. $^{*}p < .05$, $^{**}p < .01$, $^{***}p < .001$

Supplementary Table 3. Linear regression models predicting carbon footprint inequality perception with covariates by country.

	**Total**	**Denmark**	**India**	**Nigeria**	**United States**
(Intercept)	1.25 ** [0.49, 2.00]	1.31 *** [1.24, 1.37]	6.76 *** [4.82, 8.69]	1.51 *** [1.01, 2.02]	1.85 *** [1.70, 2.00]
Climate change concern	-0.21 [-0.65, 0.23]	0.01 [-0.05, 0.07]	-0.25 [-1.82, 1.32]	-0.82 *** [-1.24, -0.40]	-0.15 * [-0.29, -0.01]
Personal norm	-0.25 [-0.71, 0.21]	-0.07 * [-0.13, -0.01]	-1.44 [-3.03, 0.15]	0.54 ** [0.13, 0.94]	0.10 [-0.05, 0.25]
Descriptive norm	0.54 ** [0.16, 0.92]	0.03 [-0.01, 0.08]	1.45 * [0.06, 2.84]	0.35 [-0.03, 0.73]	0.06 [-0.05, 0.18]
Trust in government	0.39 * [0.06, 0.72]	-0.02 [-0.05, 0.02]	1.25 [-0.07, 2.57]	-0.09 [-0.44, 0.25]	0.02 [-0.07, 0.10]
Political orientation	0.59 *** [0.28, 0.90]	0.06 ** [0.02, 0.10]	1.98 ** [0.74, 3.21]	-0.02 [-0.35, 0.32]	0.05 [-0.03, 0.13]
Age	-0.26 [-0.57, 0.06]	-0.02 [-0.05, 0.02]	-0.18 [-1.36, 1.01]	-0.97 *** [-1.31, -0.62]	-0.08 * [-0.16, -0.00]
Top 10% of income	-0.19 [-0.83, 0.44]	-0.07 [-0.14, 0.01]	0.09 [-2.26, 2.44]	-0.18 [-0.89, 0.53]	-0.12 [-0.27, 0.04]
Female	0.29 [-0.35, 0.92]	0.06 [-0.02, 0.13]	-0.00 [-2.35, 2.34]	0.05 [-0.66, 0.76]	0.04 [-0.12, 0.20]
Ref. (Denmark)					
India	5.50 *** [4.64, 6.35]				
Nigeria	0.18 [-0.68, 1.05]				
United States	0.48 [-0.39, 1.35]				
Observations	3,747	923	949	956	919
R^2 / R^2 adjusted	0.065 / 0.061	0.030 / 0.021	0.030 / 0.020	0.052 / 0.043	0.020 / 0.010

Top 10% of income shows a coefficient relative to participants belonging to the 'general population' in their country, whereas Female shows a coefficient relative to identifying as male. $^{*}p < .05$, $^{**}p < .01$, $^{***}p < .001$

Supplementary Table 4. Composite climate policy support (test of hypothesis 2).

	**Without outliers (pre-registered)**		**Full sample**		**Top 10% reference**	
	Estimates	*95% CI*	*Estimates*	*95% CI*	*Estimates*	*95% CI*
(Intercept)	4.52 ***	4.14, 4.91	4.50 ***	4.13, 4.88	4.53 ***	4.16, 4.91
CF inequality perception	-0.07 ***	-0.11, -0.03	-0.03	-0.06, 0.01	-0.07 ***	-0.10, -0.03
Top 10% of income	0.08 *	0.01, 0.16	0.11 **	0.04, 0.18	0.09 *	0.01, 0.16
**Random Effects**						
σ^2	1.34		1.37		1.34	
τ_{00}	0.15 country		0.14 country		0.14 country	
ICC	0.10		0.09		0.10	
N	4 country		4 country		4 country	
Observations	3,756		4,003		3,757	
Marginal R^2 / Conditional R^2	0.004 / 0.106		0.002 / 0.096		0.004 / 0.101	

Top 10% of income shows a coefficient relative to participants belonging to the 'general population' in their country. $^{*}p < .05$, $^{**}p < .01$, $^{***}p < .001$

Supplementary Table 5. Linear regression models predicting composite climate policy support by country.

	**Total**	**Denmark**	**India**	**Nigeria**	**United States**
(Intercept)	4.37 *** [4.29, 4.45]	4.38 *** [4.27, 4.49]	4.92 *** [4.92, 5.01]	4.77 *** [4.69, 4.86]	4.02 *** [3.89, 4.15]
CF inequality perception	-0.07 *** [-0.11, -0.03]	-0.17 *** [-0.25, -0.09]	0.00 [-0.06, 0.07]	-0.12 *** [-0.18, -0.06]	0.00 [-0.09, 0.09]
Top 10% of income	0.08 * [0.01, 0.16]	0.07 [-0.08, 0.23]	0.29 *** [0.16, 0.42]	-0.03 [-0.15, 0.09]	-0.01 [-0.19, 0.17]
Ref. (Denmark)					
India	0.65 *** [0.55, 0.76]				
Nigeria	0.34 *** [0.24, 0.45]				
United States	-0.40 *** [-0.50, -0.29]				
Observations	3,756	931	949	956	920
R^2 / R^2 adjusted	0.106 / 0.105	0.020 / 0.018	0.020 / 0.018	0.015 / 0.013	0.000 / -0.002

Top 10% of income shows a coefficient relative to participants belonging to the 'general population' in their country. $^{*}p < .05$, $^{**}p < .01$, $^{***}p < .001$

Supplementary Table 6. Linear regression models predicting composite climate policy support with an interaction effect between carbon footprint inequality perception and participants' income segment.

	**Total**	**Denmark**	**India**	**Nigeria**	**United States**
(Intercept)	4.37 *** [4.29, 4.46]	4.37 *** [4.26, 4.49]	4.92 *** [4.83, 5.01]	4.77 *** [4.69, 4.86]	4.02 *** [3.89, 4.15]
CF inequality perception	-0.09 *** [-0.13, -0.04]	-0.10 * [-0.20, -0.01]	-0.01 [-0.11, 0.09]	-0.15 *** [-0.21, -0.08]	-0.05 [-0.17, 0.07]
Top 10% of income	0.09 * [0.01, 0.16]	0.07 [-0.09, 0.23]	0.29 *** [0.16, 0.42]	-0.03 [-0.15, 0.09]	-0.01 [-0.19, 0.18]
CF inequality perception × Top 10% of income	0.05 [-0.03, 0.12]	-0.18 * [-0.35, -0.02]	0.03 [-0.11, 0.16]	0.13 [-0.02, 0.28]	0.12 [-0.07, 0.30]
Ref. (Denmark)					
India	0.65 *** [0.55, 0.76]				
Nigeria	0.34 *** [0.24, 0.45]				
United States	-0.40 *** [-0.50, -0.29]				
Observations	3,756	931	949	956	920
R^2 / R^2 adjusted	0.107 / 0.105	0.026 / 0.022	0.020 / 0.017	0.018 / 0.015	0.002 / -0.002

Top 10% of income shows a coefficient relative to participants belonging to the 'general population' in their country. $^{*}p < .05$, $^{**}p < .01$, $^{***}p < .001$

Supplementary Table 7. Mixed-effects model predicting composite climate policy support with covariates

	Climate policy support	
	Estimates	*95% CI*
(Intercept)	4.38 ***	4.30, 4.45
CF inequality perception	-0.05 ***	-0.08, -0.02
Climate change concern	0.40 ***	0.36, 0.44
Personal norm	0.23 ***	0.19, 0.28
Descriptive norm	0.08 ***	0.04, 0.12
Trust in government	0.18 ***	0.15, 0.21
Political orientation	-0.11 ***	-0.14, -0.08
Age	0.02	-0.01, 0.05
Top 10% of income	0.06 *	0.00, 0.12
Female	0.00	-0.06, 0.07
Ref. (Denmark)		
India	0.66 ***	0.58, 0.74
Nigeria	0.34 ***	0.26, 0.42
United States	-0.38 ***	-0.47, -0.30
**Random Effects**		
σ^2	2.66	
τ_{00} ResponseId	0.60	
ICC	0.19	
N ResponseId	3,747	
Observations	44,964	
Marginal R^2 / Conditional R^2	0.171 / 0.324	

Top 10% of income shows a coefficient relative to participants belonging to the 'general population' in their country, whereas Female shows a coefficient relative to identifying as male. $^{*}p < .05$, $^{**}p < .01$, $^{***}p < .001$

Supplementary Table 8. Linear regression models predicting composite support for climate policies with covariates by country

	**Total**	**Denmark**	**India**	**Nigeria**	**United States**
(Intercept)	4.38 *** [4.30, 4.45]	4.33 *** [4.24, 4.42]	5.01 *** [4.91, 5.11]	4.83 *** [4.74, 4.92]	4.06 *** [3.93, 4.18]
CF inequality perception	-0.05 *** [-0.08, -0.02]	-0.06 * [-0.12, -0.01]	-0.02 [-0.08, 0.03]	-0.09 ** [-0.14, -0.03]	-0.01 [-0.07, 0.05]
Climate change concern	0.40 *** [0.36, 0.44]	0.52 *** [0.43, 0.60]	0.18 *** [0.10, 0.26]	0.25 *** [0.18, 0.32]	0.61 *** [0.49, 0.72]
Personal norm	0.23 *** [0.19, 0.28]	0.29 *** [0.20, 0.38]	0.19 *** [0.11, 0.27]	0.13 *** [0.06, 0.20]	0.17 ** [0.04, 0.29]
Descriptive norm	0.08 *** [0.04, 0.12]	0.00 [-0.06, 0.06]	0.06 [-0.01, 0.13]	0.10 ** [0.03, 0.16]	0.14 ** [0.05, 0.23]
Trust in government	0.18 *** [0.15, 0.21]	0.17 *** [0.11, 0.22]	0.07 * [0.00, 0.14]	0.06 [-0.00, 0.12]	0.23 *** [0.15, 0.30]
Political orientation	-0.11 *** [-0.14, -0.08]	-0.20 *** [-0.26, -0.15]	0.13 *** [0.07, 0.19]	0.04 [-0.02, 0.10]	-0.12 *** [-0.19, -0.06]
Age	0.02 [-0.01, 0.05]	-0.03 [-0.08, 0.03]	0.13 *** [0.07, 0.19]	0.19 *** [0.13, 0.26]	-0.12 *** [-0.18, -0.06]
Top 10% of income	0.06 * [0.00, 0.12]	0.10 [-0.01, 0.21]	0.14 * [0.03, 0.26]	-0.05 [-0.17, 0.08]	-0.07 [-0.19, 0.06]
Female	0.36 [-0.06, 0.07]	0.06 [-0.05, 0.17]	-0.04 [-0.16, 0.08]	-0.21 *** [-0.34, -0.09]	0.03 [-0.66, 0.72]
Ref. (Denmark)					
India	0.66 *** [0.57, 0.74]				
Nigeria	0.34 *** [0.26, 0.42]				
United States	-0.38 *** [-0.47, -0.30]				
Observations	3,747	923	949	956	919
R^2 / R^2 adjusted	0.449 / 0.447	0.569 / 0.564	0.245 / 0.236	0.254 / 0.246	0.577 / 0.573

Top 10% of income shows a coefficient relative to participants belonging to the 'general population' in their country, whereas Female shows a coefficient relative to identifying as male. $^{*}p < .05$, $^{**}p < .01$, $^{***}p < .001$

Supplementary Table 9. Perceived fairness of actual carbon footprint inequality (test of hypothesis 3).

	**Without outliers (pre-registered)**		**Full sample**		**Top 10% reference**	
	Estimates	*95% CI*	*Estimates*	*95% CI*	*Estimates*	*95% CI*
(Intercept)	3.43 ***	3.00, 3.87	3.45 ***	3.02, 3.88	3.43 ***	2.99, 3.87
CF inequality perception	0.16 ***	0.10, 0.22	0.09 **	0.03, 0.14	0.19 ***	0.13, 0.25
Top 10% of income	0.44 ***	0.32, 0.56	0.43 ***	0.31, 0.54	0.44 ***	0.32, 0.56
**Random Effects**						
σ^2	3.45		3.47		3.44	
τ_{00}	0.19_{country}		0.19_{country}		0.19_{country}	
ICC	0.05		0.05		0.05	
N	4_{country}		4_{country}		4_{country}	
Observations	3,756		4,003		3,757	
Marginal R^2 / Conditional R^2	0.020 / 0.071		0.014 / 0.064		0.022 / 0.074	

Top 10% of income shows a coefficient relative to participants belonging to the 'general population' in their country. $^{*}p < .05$, $^{**}p < .01$, $^{***}p < .001$

Supplementary Table 10. Linear regression models predicting perceived fairness of actual carbon footprint inequality by country.

	Total	Denmark	India	Nigeria	United States
(Intercept)	2.89 *** [2.75, 3.02]	2.88 *** [2.73, 3.03]	3.89 *** [3.70, 4.08]	3.86 *** [3.70, 4.02]	3.12 *** [2.95, 3.29]
CF inequality perception	0.16 *** [0.10, 0.22]	0.06 [-0.05, 0.17]	0.36 *** [0.23, 0.50]	0.09 [-0.02, 0.20]	0.15 * [0.03, 0.27]
Top 10% of income	0.44 *** [0.32, 0.56]	0.46 *** [0.25, 0.67]	0.20 [-0.06, 0.47]	0.62 *** [0.39, 0.84]	0.46 *** [0.22, 0.71]
Ref. (Denmark)					
India	0.88 *** [0.72, 1.05]				
Nigeria	1.07 *** [0.90, 1.23]				
United States	0.24 ** [0.07, 0.41]				
Observations	3,756	931	949	956	920
R^2 / R^2 adjusted	0.072 / 0.070	0.020 / 0.018	0.032 / 0.030	0.031 / 0.029	0.020 / 0.018

Top 10% of income shows a coefficient relative to participants belonging to the 'general population' in their country. $^{*}p < .05$, $^{**}p < .01$, $^{***}p < .001$

Supplementary Table 11. Linear regression models predicting perceived fairness actual carbon footprint inequality with an interaction effect between carbon footprint inequality perception and participants' income segment.

	**Total**	**Denmark**	**India**	**Nigeria**	**United States**
(Intercept)	2.89 *** [2.76, 3.02]	2.88 *** [2.73, 3.03]	3.89 *** [3.70, 4.08]	3.87 *** [3.71, 4.02]	3.12 *** [2.95, 3.30]
CF inequality perception	0.09 * [0.01, 0.16]	0.04 [-0.09, 0.18]	0.25 * [0.05, 0.45]	0.06 [-0.07, 0.18]	0.06 [-0.10, 0.22]
Top 10% of income	0.45 *** [0.33, 0.56]	0.46 *** [0.25, 0.67]	0.20 [-0.06, 0.47]	0.62 *** [0.40, 0.85]	0.47 *** [0.22, 0.71]
CF inequality perception × Top 10% of income	0.19 ** [0.07, 0.31]	0.04 [-0.18, 0.26]	0.21 [-0.06, 0.47]	0.15 [-0.13, 0.42]	0.20 [-0.05, 0.45]
Ref. (Denmark)					
India	0.88 *** [0.71, 1.05]				
Nigeria	1.07 *** [0.90, 1.23]				
United States	0.24 ** [0.08, 0.41]				
Observations	3,756	931	949	956	920
R^2 / R^2 adjusted	0.074 / 0.073	0.020 / 0.017	0.034 / 0.031	0.032 / 0.029	0.023 / 0.019

Top 10% of income shows a coefficient relative to participants belonging to the 'general population' in their country. $^{*}p < .05$, $^{**}p < .01$, $^{***}p < .001$

Supplementary Table 12. Mixed-effects model predicting perceived fairness of actual carbon footprint inequality with covariates.

	Perceived fairness	
	Estimates	*95% CI*
(Intercept)	3.59 ***	3.16, 4.02
CF inequality perception	0.10 ***	0.05, 0.16
Climate change concern	-0.13 **	-0.21, -0.05
Personal norm	-0.06	-0.14, 0.03
Descriptive norm	0.21 ***	0.14, 0.28
Trust in government	0.31 ***	0.25, 0.37
Political orientation	0.46 ***	0.41, 0.52
Age	-0.09 **	-0.15, -0.03
Top 10% of income	0.28 ***	0.16, 0.39
Female	-0.17 **	-0.28, -0.05
**Random Effects**		
σ^2	3.03	
$\tau_{00\ country}$	0.18	
ICC	0.06	
$N_{country}$	4	
Observations	3,747	
Marginal R^2 / Conditional R^2	0.132 / 0.181	

Top 10% of income shows a coefficient relative to participants belonging to the 'general population' in their country, whereas Female shows a coefficient relative to identifying as male. $^{*}p < .05$, $^{**}p < .01$, $^{***}p < .001$

Supplementary Table 13. Linear regression models predicting perceived fairness of actual carbon footprint inequality with covariates by country.

	**Total**	**Denmark**	**India**	**Nigeria**	**United States**
(Intercept)	3.04 *** [2.90, 3.18]	3.00 *** [2.83, 3.17]	3.89 *** [3.68, 4.10]	4.04 *** [3.87, 4.21]	3.34 *** [3.11, 3.57]
CF inequality perception	0.10 *** [0.05, 0.16]	-0.02 [-0.12, 0.08]	0.24 *** [0.11, 0.37]	0.11 * [0.00, 0.21]	0.08 [-0.04, 0.19]
Climate change concern	-0.13 ** [-0.21, -0.05]	-0.25 ** [-0.41, -0.09]	-0.06 [-0.23, 0.11]	0.20 ** [0.05, 0.34]	-0.37 *** [-0.58, -0.16]
Personal norm	-0.06 [-0.14, 0.03]	-0.10 [-0.27, 0.06]	-0.15 [-0.32, 0.02]	0.16 * [0.03, 0.30]	-0.04 [-0.27, 0.20]
Descriptive norm	0.21 *** [0.14, 0.28]	0.08 [-0.04, 0.20]	0.38 *** [0.23, 0.53]	0.09 [-0.04, 0.22]	0.21 * [0.04, 0.38]
Trust in government	0.31 *** [0.25, 0.37]	0.15 ** [0.05, 0.25]	0.36 *** [0.22, 0.50]	0.38 *** [0.26, 0.49]	0.39 *** [0.26, 0.53]
Political orientation	0.46 *** [0.41, 0.52]	0.41 *** [0.30, 0.52]	0.34 *** [0.20, 0.47]	0.24 *** [0.13, 0.35]	0.53 *** [0.40, 0.65]
Age	-0.09 ** [-0.15, -0.03]	-0.25 *** [-0.35, -0.15]	-0.03 [-0.16, 0.10]	0.12 * [0.00, 0.24]	-0.23 *** [-0.35, -0.12]
Top 10% of income	0.28 *** [0.16, 0.39]	0.40 *** [0.20, 0.61]	0.07 [-0.19, 0.32]	0.49 *** [0.25, 0.73]	0.24 [-0.00, 0.48]
Female	-0.16 ** [-0.28, -0.05]	-0.21 * [-0.41, -0.01]	0.20 [-0.05, 0.46]	-0.47 *** [-0.71, -0.24]	-0.20 [-0.44, 0.05]
Ref. (Denmark)					
India	0.90 *** [0.74, 1.06]				
Nigeria	1.04 *** [0.88, 1.20]				
United States	0.27 ** [0.11, 0.43]				
Observations	3,747	923	949	956	919
R^2 / R^2 adjusted	0.183 / 0.180	0.163 / 0.153	0.155 / 0.145	0.209 / 0.200	0.191 / 0.182

Top 10% of income shows a coefficient relative to participants belonging to the 'general population' in their country, whereas Female shows a coefficient relative to identifying as male. $^{*}p < .05$, $^{**}p < .01$, $^{***}p < .001$

Supplementary Table 14. Sensitivity analysis of relative estimation error associated with estimated personal carbon footprints with additional outlier removal (*H1*).

	Sensitivity analysis	
	Estimates	*95% CI*
(Intercept)	1.45 ***	0.94, 1.95
Estimation error (Top 1%)	-2.21 ***	-2.36, -2.05
Estimation error (Top 10%)	-1.70 ***	-1.85, -1.55
Income segment (Top 10%)	0.07	-0.06, 0.20
**Random Effects**		
σ^2	11.20	
$\tau_{00\ \mathrm{ResponseId}}$	0.27	
$\tau_{00\ \mathrm{country}}$	0.25	
ICC	0.04	
N_{country}	4	
$N_{\mathrm{ResponseId}}$	3,943	
Observations	11,302	
Marginal R^2 / Conditional R^2	0.071 / 0.112	

Income segment (Top 10%) refers to participants belonging to the Top 10% of income within their country and shows coefficients relative to belonging to the general population. $^{*}p < .05$, $^{**}p < .01$, $^{***}p < .001$

Supplementary Table 15. Sensitivity analysis of linear regression models predicting carbon footprint inequality perception with covariates by country with additional outlier removal (+/- 3SD).

	Total	Denmark	India	Nigeria	United States
(Intercept)	1.18 *** [0.75, 1.62]	1.25 *** [1.20, 1.30]	4.86 *** [3.74, 5.99]	0.64 *** [0.43, 0.86]	1.71 *** [1.59, 1.84]
Climate change concern	-0.10 [-0.35, 0.15]	-0.02 [-0.06, 0.03]	-0.27 [-1.19, 0.64]	-0.12 [-0.30, 0.06]	-0.02 [-0.14, 0.09]
Personal norm	-0.28 * [-0.54, -0.01]	-0.03 [-0.08, 0.01]	-0.91 [-1.85, 0.03]	0.02 [-0.15, 0.19]	-0.01 [-0.13, 0.12]
Descriptive norm	0.41 *** [0.19, 0.62]	0.03 [-0.00, 0.07]	1.06 * [0.25, 1.87]	0.26 ** [0.10, 0.42]	0.03 [-0.06, 0.13]
Trust in government	0.20 * [0.01, 0.39]	-0.00 [-0.04, 0.03]	0.80 * [0.03, 1.56]	-0.20 ** [-0.34, -0.05]	0.04 [-0.04, 0.11]
Political orientation	0.18 * [0.01, 0.36]	0.06 *** [0.03, 0.09]	0.47 [-0.25, 1.20]	-0.09 [-0.23, 0.05]	0.06 [-0.00, 0.13]
Age	-0.09 [-0.27, 0.09]	-0.00 [-0.03, 0.03]	0.13 [-0.56, 0.82]	-0.57 *** [-0.72, -0.43]	-0.05 [-0.11, 0.01]
Top 10% of income	-0.25 [-0.61, 0.11]	-0.02 [-0.08, 0.04]	-1.00 [-2.37, 0.37]	0.54 *** [0.24, 0.83]	-0.07 [-0.20, 0.05]
Female	0.43 * [0.07, 0.80]	0.06 * [0.00, 0.12]	0.88 [-0.48, 2.25]	0.23 [-0.07, 0.53]	0.10 [-0.03, 0.23]
Ref. (Denmark)					
India	3.45 *** [2.96, 3.94]				
Nigeria	-0.22 [-0.71, 0.27]				
United States	0.42 [-0.07, 0.91]				
Observations	3,640	896	912	939	893
R^2 / R^2 adjusted	0.082 / 0.078	0.037 / 0.027	0.028 / 0.017	0.081 / 0.072	0.016 / 0.006

Top 10% of income shows a coefficient relative to participants belonging to the 'general population' in their country, whereas Female shows a coefficient relative to identifying as male. $^{*}p < .05$, $^{**}p < .01$, $^{***}p < .001$

Supplementary Fig. 1. Support for individual climate policies (Denmark). Results of linear regression models predicting support for individual climate policies. All covariates were standardized, except 'Top 10% of income' and Female (see Methods). Female shows a coefficient relative to identifying as male. Dots represent point estimates with 95% confidence intervals. The sample size is $N = 923$ for all policies.

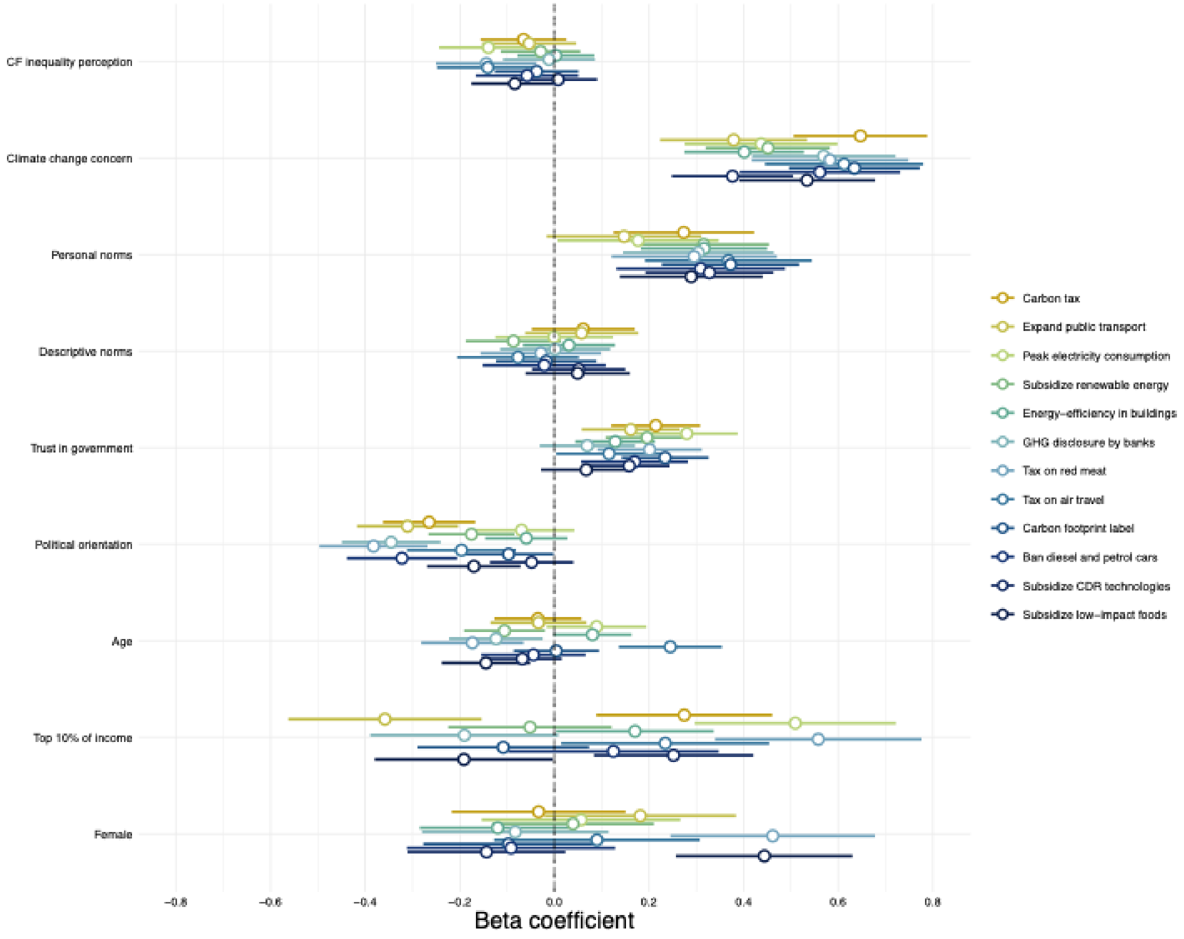

Supplementary Fig. 2. Support for individual climate policies (India). Results of linear regression models predicting support for individual climate policies. All covariates were standardized, except ‘Top 10% of income’ and Female (see Methods). Female shows a coefficient relative to identifying as male. Dots represent point estimates with 95% confidence intervals. The sample size is $N = 949$ for all policies.

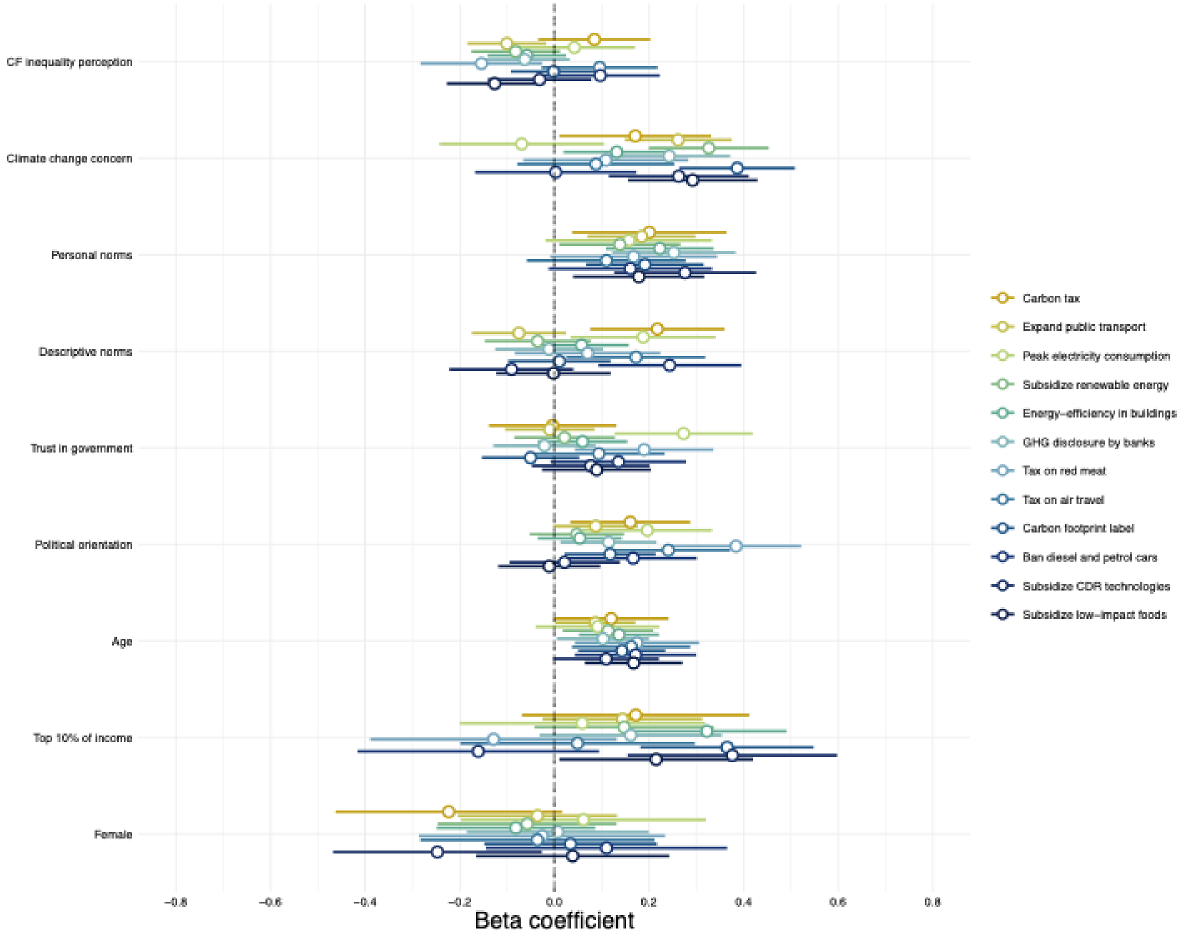

Supplementary Fig. 3. Support for individual climate policies (Nigeria). Results of linear regression models predicting support for individual climate policies. All covariates were standardized, except 'Top 10% of income' and Female (see Methods). Female shows a coefficient relative to identifying as male. Dots represent point estimates with 95% confidence intervals. The sample size is $N = 956$ for all policies.

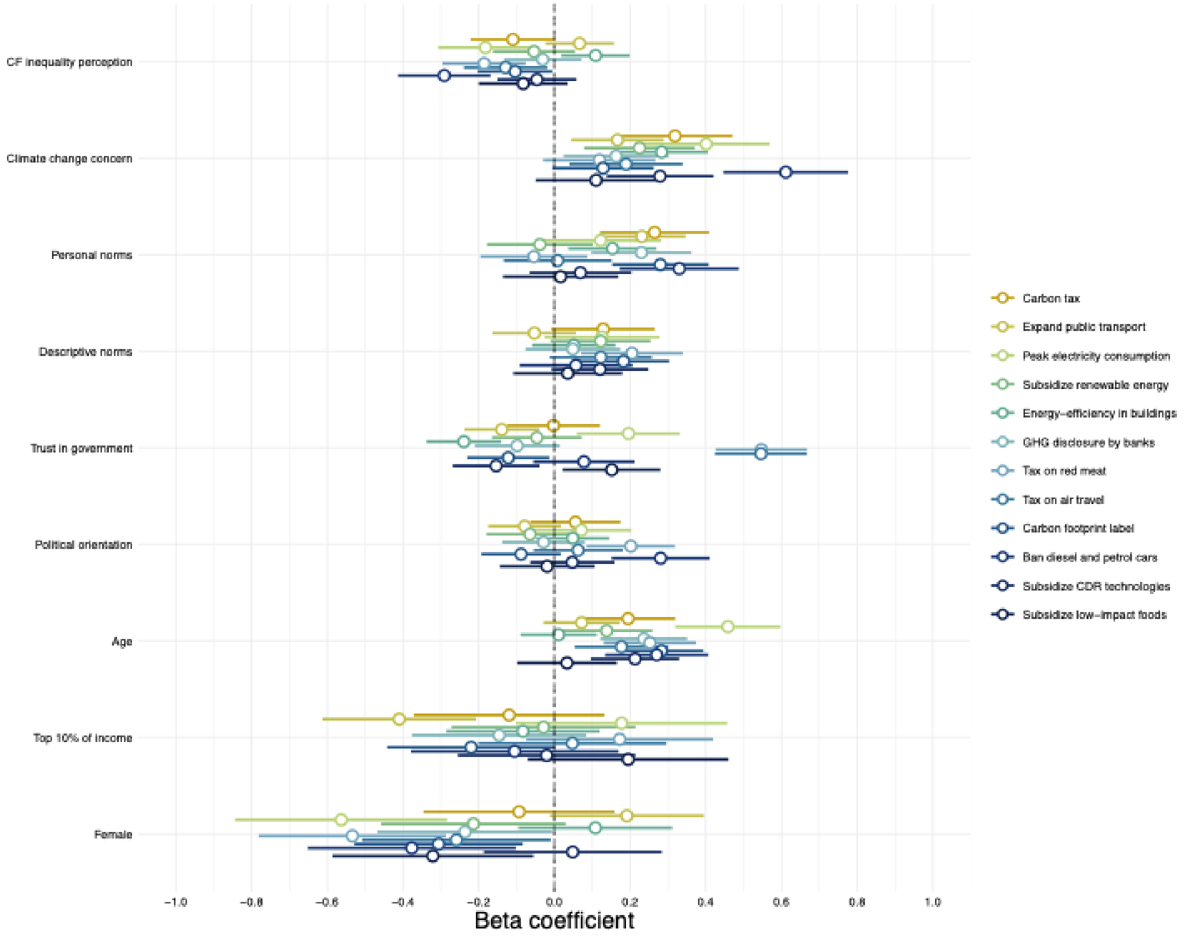

Supplementary Fig. 4. Support for individual climate policies (United States). Results of linear regression models predicting support for individual climate policies. All covariates were standardized, except 'Top 10% of income' and Female (see Methods). Female shows a coefficient relative to identifying as male. Dots represent point estimates with 95% confidence intervals. The sample size is $N = 919$ for all policies.

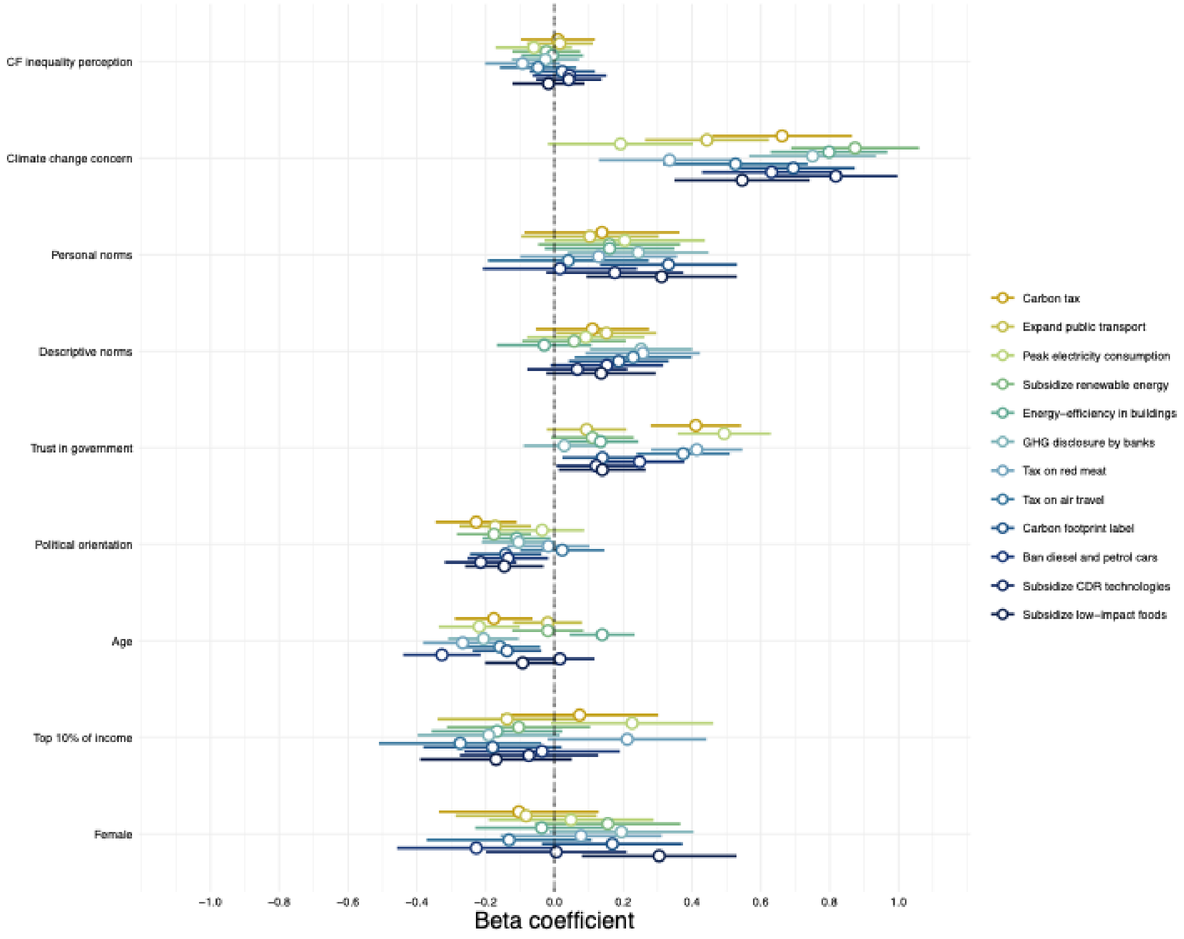

Supplementary Fig. 5. Sensitivity analysis with additional outlier removal from relative estimation error variables and carbon footprint inequality perception. **A**, relative estimation error for the Bottom 50% of income with additional outlier removal (+/- 3SD from the pre-registered 2.5-97.5 percentiles per income group and country). **B**, relative estimation error for the Top 10% of income (+/- 3SD from the pre-registered 2.5-97.5 percentiles per income group and country). **C**, relative estimation error for the Top 1% of income (+/- 3SD from the pre-registered 2.5-97.5 percentiles per income group and country). **D**, carbon footprint inequality perception with additional outlier removal. Positive values reflect an underestimation of the average carbon footprint of the Top 1% income group relative to the Bottom 50% income group. In contrast, negative values indicate an overestimation of the average carbon footprint of the Top 1 % income group relative to the Bottom 50% income group. **E**, carbon footprint inequality perception z-standardized at the country level.

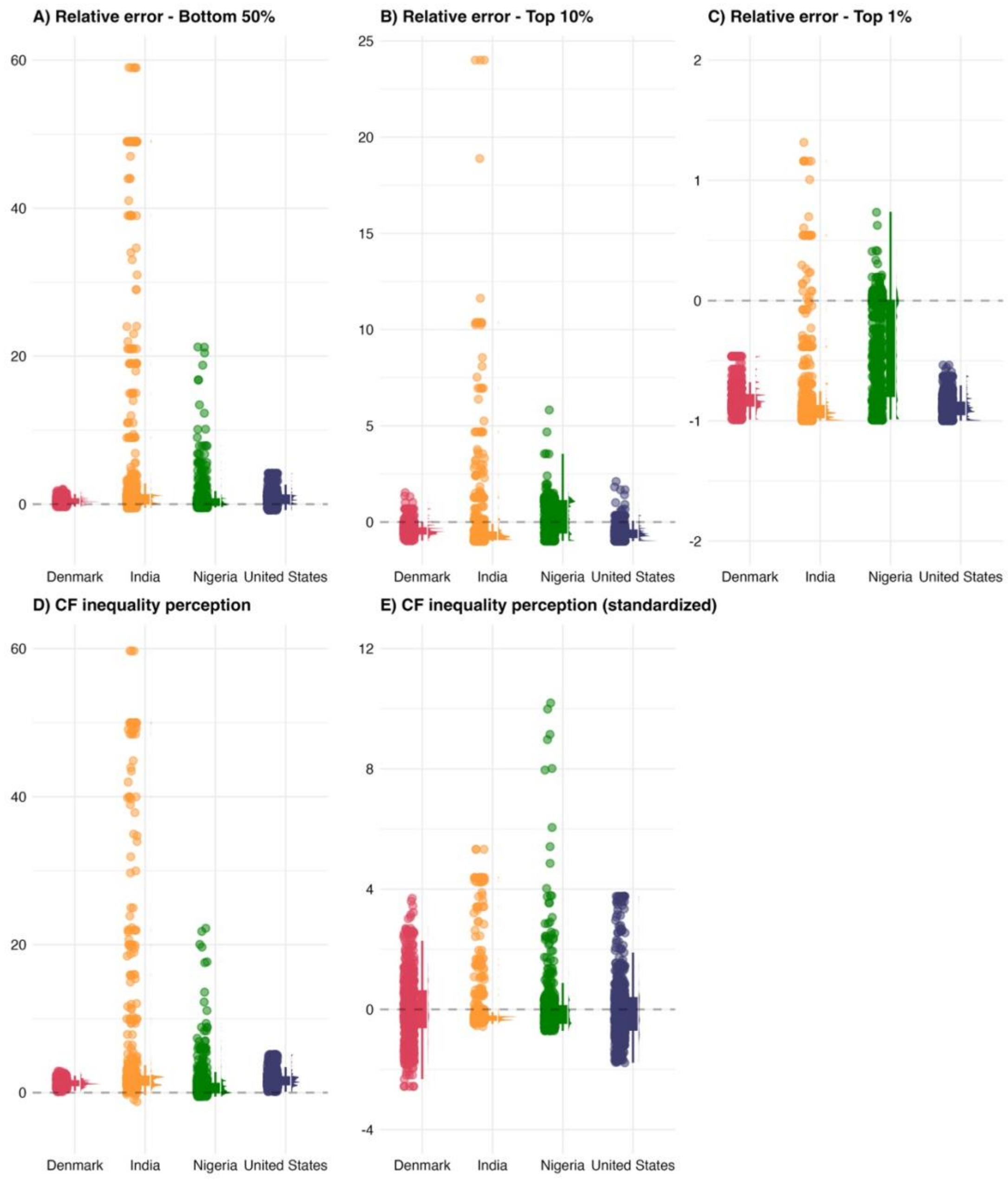

Supplementary Fig. 6. Sensitivity analysis of predictors of composite climate policy support with additional outlier removal (+/- 3SD; *H2*). A, Mixed-effects regression model predicting composite climate policy support at the aggregate level (*N* = 3,649). **B**, Country-specific linear regression models predicting composite climate policy support. The sample sizes are Denmark (*N* = 904), India (*N* = 912), Nigeria (*N* = 939), and the United States (*N* = 894). **C**, Country-specific linear regression models predicting composite climate policy support with socio-demographic and psychological covariates. All covariates were standardized at the country level, except for Top 10% of income and Female (see Methods). Top 10% of income shows a coefficient relative to participants belonging to the 'general population' in their country, whereas Female shows a coefficient relative to identifying as male. The sample sizes are Denmark (*N* = 896), India (*N* = 912), Nigeria (*N* = 939), and the United States (*N* = 893). Dots represent point estimates with 95% confidence intervals.

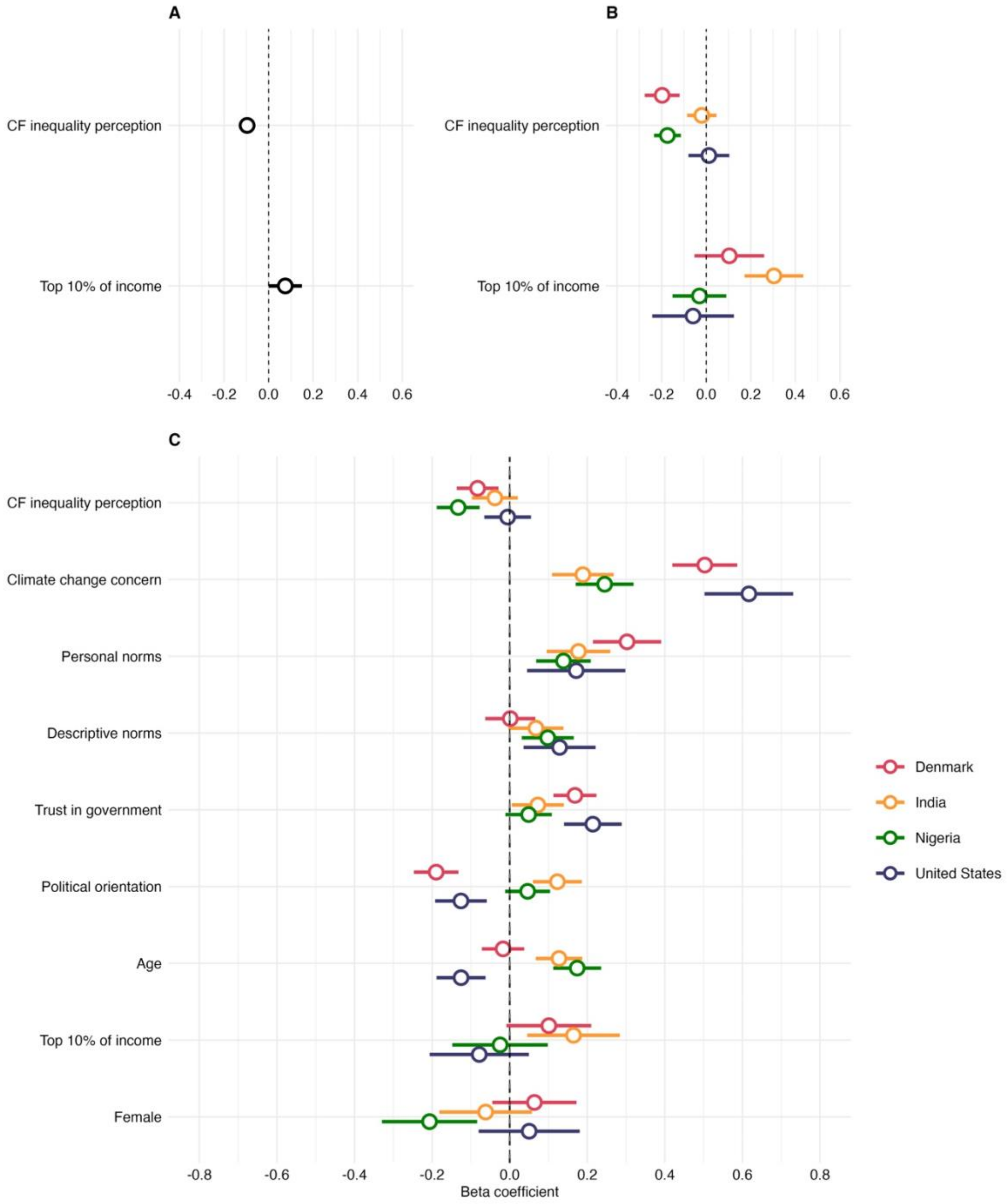

Supplementary Fig. 7. Sensitivity analysis of perceived fairness of actual carbon footprint inequality with additional outlier removal (+/- 3SD; *H3*). A, Mixed-effects regression model predicting perceived fairness of actual carbon footprint inequality at the aggregate level (*N* = 3,649). **B**, Country-specific linear regression models predicting perceived fairness of actual carbon footprint inequality. The sample sizes are Denmark (*N* = 904), India (*N* = 912), Nigeria (*N* = 939), and the United States (*N* = 894). **C**, Country-specific linear regression models predicting perceived fairness of actual carbon footprint inequality with socio-demographic and psychological covariates. All covariates were standardized at the country level, except for Top 10% of income and Female (see Methods). Top 10% of income shows a coefficient relative to participants belonging to the 'general population' in their country, whereas Female shows a coefficient relative to identifying as male. The sample sizes are Denmark (*N* = 896), India (*N* = 912), Nigeria (*N* = 939), and the United States (*N* = 893). Dots represent point estimates with 95% confidence intervals.

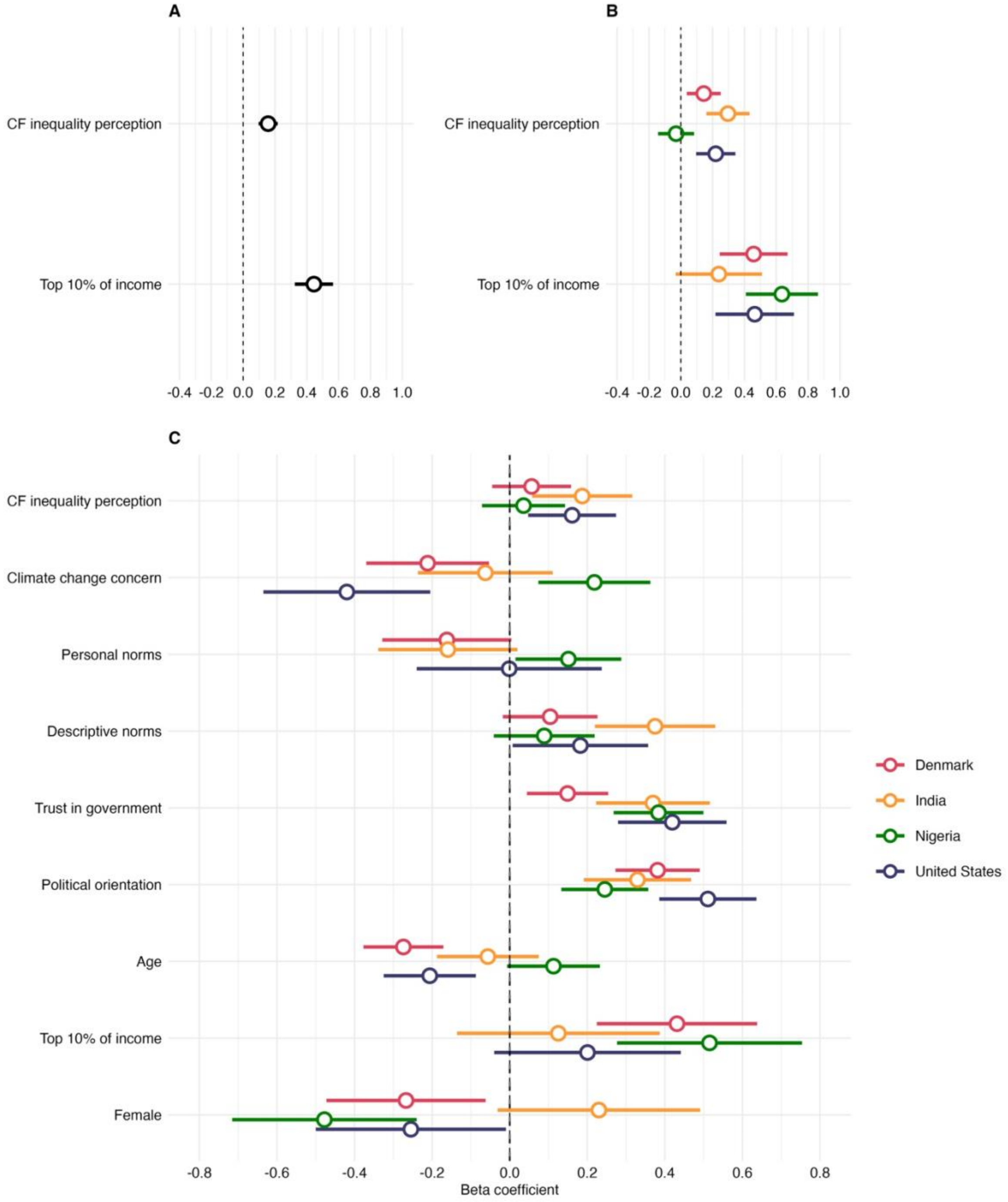

Supplement: Supplementary file 1 — Supplementary Tables 1–15 and Figs. 1–7. [file 41558_2024_2130_MOESM1_ESM.pdf]
