# Supplementary material for: Underestimation of personal carbon footprint inequality in four diverse countries
Source: Nat Clim Chang. 2024 Sep 12;14(11):1136–43. doi: 10.1038/s41558-024-02130-y (PMC11537954; doi:10.1038/s41558-024-02130-y)
Supplement: Supplementary file 2 — Reporting Summary [file 41558_2024_2130_MOESM2_ESM.pdf]

nature portfolio

Corresponding author(s): Kristian Steensen Nielsen

Last updated by author(s): Aug 9, 2024

Reporting Summary

Nature Portfolio wishes to improve the reproducibility of the work that we publish. This form provides structure for consistency and transparency in reporting. For further information on Nature Portfolio policies, see our Editorial Policies and the Editorial Policy Checklist.

Statistics

For all statistical analyses, confirm that the following items are present in the figure legend, table legend, main text, or Methods section.

n/a	Confirmed	
☐	☒	The exact sample size (*n*) for each experimental group/condition, given as a discrete number and unit of measurement
☐	☒	A statement on whether measurements were taken from distinct samples or whether the same sample was measured repeatedly
☐	☒	The statistical test(s) used AND whether they are one- or two-sided<br>*Only common tests should be described solely by name; describe more complex techniques in the Methods section.*
☐	☒	A description of all covariates tested
☐	☒	A description of any assumptions or corrections, such as tests of normality and adjustment for multiple comparisons
☐	☒	A full description of the statistical parameters including central tendency (e.g. means) or other basic estimates (e.g. regression coefficient) AND variation (e.g. standard deviation) or associated estimates of uncertainty (e.g. confidence intervals)
☐	☒	For null hypothesis testing, the test statistic (e.g. *F*, *t*, *r*) with confidence intervals, effect sizes, degrees of freedom and *P* value noted<br>*Give P values as exact values whenever suitable.*
☒	☐	For Bayesian analysis, information on the choice of priors and Markov chain Monte Carlo settings
☒	☐	For hierarchical and complex designs, identification of the appropriate level for tests and full reporting of outcomes
☐	☒	Estimates of effect sizes (e.g. Cohen's *d*, Pearson's *r*), indicating how they were calculated

Our web collection on statistics for biologists contains articles on many of the points above.

Software and code

Policy information about availability of computer code

Data collection	The data was collected through online surveys conducted in Denmark, India, Nigeria, and the United States via Qualtrics and Nielsen. We used Qualtrics' software (version: December, 2023) to programme and execute the survey.
Data analysis	For the data analyses, we used the following R packages: jtools (v. 2.2.2), lme4 (v. 1.1-35.3), psych (v. 2.4.6.26), patchwork (v. 1.2.0), ggplot2 (v. 3.5.1), sjPlot (v. 2.8.16), viridis (v. 0.6.5), and RColorBrewer (v. 1.1-3)

For manuscripts utilizing custom algorithms or software that are central to the research but not yet described in published literature, software must be made available to editors and reviewers. We strongly encourage code deposition in a community repository (e.g. GitHub). See the Nature Portfolio guidelines for submitting code & software for further information.

Data

Policy information about availability of data

All manuscripts must include a data availability statement. This statement should provide the following information, where applicable:

- Accession codes, unique identifiers, or web links for publicly available datasets
- A description of any restrictions on data availability
- For clinical datasets or third party data, please ensure that the statement adheres to our policy

The data and study materials are publicly available via the Open Science Framework: https://doi.org/10.17605/OSF.IO/8QTFY. We used data from the World Inequality Database (https://wid.world/data/) to estimate the personal carbon footprints, which was extracted in May 2023:

Human research participants

Policy information about studies involving human research participants and Sex and Gender in Research.

Reporting on sex and gender	Participants self-reported the gender they identified with. We included gender as a covariate in all statistical analyses, as gender differences are commonly observed in studies on climate-related perceptions, policy support, and fairness perceptions.
Population characteristics	The study consisted of participants from four countries. A total of 4,003 participants completed the survey with the following country breakdown: Denmark (n = 1,001), India (n = 1,001), Nigeria (n = 1,001), and the United States (n = 1,000). In Denmark, the average age was 52.80 and 54% of participants were male, 46% female, and 0.1% non-binary. In India, the average age was 31.1 and 59% of participants were male, 41% female, and 0.2% non-binary. In Nigeria, the average age was 35.4 and 74% of participants were male, 26% female, and 0.1% non-binary. In the United States, the average age was 50.07 and 41% of participants were male, 58% female, and 0.9% non-binary.
Recruitment	Participants were recruited via the market research companies Nielsen (Denmark and Nigeria) and Qualtrics (India and the United States) using their online survey panels. All participants consented to participate before completing the survey and received financial compensation for their participation. In each country, the sampled participants were equally split across two income groups: participants whose personal income fell into the top 10% income bracket (Top 10% segment) and participants whose personal income was below the threshold for the top 10% income bracket (general population segment).<br><br>Due to our sampling strategy, the samples are not socio-demographically representative of the respective countries. The market research companies' (or their partners') online panels likely influenced the representativeness of the samples, especially at the highest and lowest end of the income distribution. Some religious or ethnic communities may also be under-represented.
Ethics oversight	The survey was approved by the ethics committee at the Faculty of Psychology, University of Basel (020-22-1)

Note that full information on the approval of the study protocol must also be provided in the manuscript.

Field-specific reporting

Please select the one below that is the best fit for your research. If you are not sure, read the appropriate sections before making your selection.

☐ Life sciences ☒ Behavioural & social sciences ☐ Ecological, evolutionary & environmental sciences

For a reference copy of the document with all sections, see nature.com/documents/nr-reporting-summary-flat.pdf

Behavioural & social sciences study design

All studies must disclose on these points even when the disclosure is negative.

Study description	This is a quantitative cross-sectional survey study conducted online. The survey included questions about climate policy support, perceived behavioral plasticity, a carbon footprint inequality task, psychological and social factors, and socio-demographic characteristics.
Research sample	A total of 4,003 participants completed the survey with the following country breakdown: Denmark (n = 1,001), India (n = 1,001), Nigeria (n = 1,001), and the United States (n = 1,000). We selected these countries not only due to their varying levels of actual carbon footprint inequality and average personal carbon footprints but also economic inequality. In each country, the sampled participants were equally split across two income groups: participants whose personal income fell into the top 10% income bracket (Top 10% segment) and participants whose personal income was below the threshold for the top 10% income bracket (general population segment). We applied the following income thresholds for the Top 10%: Denmark (650.000 DKK), India (₹300,000), Nigeria (₦35,000,000), and the United States ($130,000) to account for the within-country differences in income level. Due to the deliberate oversampling of participants from the top 10% income segment, our country samples are not fully representative of the full populations. The mean age in the total sample was 42.35 (SD = 16.59), and 56.8% identified as male, 42.8% as female, 0.3% as non-binary, and .01% preferred not to say.
Sampling strategy	The country-specific sample sizes were determined based on a trade-off between the costs of obtaining responses and statistical power to detect small effects in most of the planned analyses. We used a quota-based sampling strategy to over-sample participants from the top 10% income segment. This sampling strategy allowed us to investigate the perceptions of high-income individuals who, despite their disproportionately large carbon footprints, are systematically underrepresented in survey-based and behavioral science research.
Data collection	Data were collected online and self-reported by participants through a survey.
Timing	The data was collected between May and June 2023.

Data exclusions	Participants were automatically screened out if one or more of the following pre-registered criteria were met: (1) reported being under 18 years old; (2) felt uncomfortable answering the survey in English (only in India); (3) reported 'prefer not to answer' on the income question; (4) failed the attention check; (5) answered the comprehension check for the concept of personal carbon footprint wrongly twice. Additionally, participants who were likely bots based on Recaptcha score (n = 10) or completed the survey unreasonably quickly (n = 180) or more than once (n = 1) were excluded and replaced with other responses by Nielsen and Qualtrics (not pre-registered).
Non-participation	92 participants declined to give their consent and were consequently automatically excluded from the survey.
Randomization	The study did not include any experimental conditions.

Reporting for specific materials, systems and methods

We require information from authors about some types of materials, experimental systems and methods used in many studies. Here, indicate whether each material, system or method listed is relevant to your study. If you are not sure if a list item applies to your research, read the appropriate section before selecting a response.

Materials & experimental systems

n/a	Involved in the study	
☒	☐	Antibodies
☒	☐	Eukaryotic cell lines
☒	☐	Palaeontology and archaeology
☒	☐	Animals and other organisms
☒	☐	Clinical data
☒	☐	Dual use research of concern

Methods

n/a	Involved in the study	
☒	☐	ChIP-seq
☒	☐	Flow cytometry
☒	☐	MRI-based neuroimaging
